# Cingulate Gradient Dysfunction in End‐Stage Renal Disease: Associations With Clinical Phenotypes and Exploratory Transcriptomic Signatures

**DOI:** 10.1002/cns.70976

**Published:** 2026-07-01

**Authors:** Peng Li, Jun‐Ya Mu, Xin‐Yi Zhu, Hui‐Jie Yuan, Zhao‐Yao Luo, Qian‐Ge Zhu, Xuan Niu, Xiu‐Long Feng, Yu Han, Ting Ge, Chen‐Xi Wang, Wen Wang, Ming Zhang

**Affiliations:** ^1^ Department of Medical Imaging The First Affiliated Hospital of Xi'an Jiaotong University Xi'an Shaanxi China; ^2^ Department of Medical Imaging Nuclear Industry 215 Hospital of Shaanxi Province Xianyang Shaanxi China; ^3^ Department of Radiology & Functional and Molecular Imaging Key Lab of Shaanxi Province, Tangdu Hospital Fourth Military Medical University Xi'an Shaanxi China

**Keywords:** cingulate cortex, end‐stage renal disease, functional magnetic resonance imaging, gene expression, gradient

## Abstract

**Aims:**

The cingulate cortex is highly vulnerable in end‐stage renal disease (ESRD) patients, but whether ESRD disrupts its functional gradient and the links to clinical phenotypes and underlying molecular mechanisms remain unclear.

**Methods:**

We prospectively enrolled clinical and resting‐state functional MRI data from 125 participants (77 ESRD patients, 48 healthy controls) to explore cingulate gradient alterations. Associations between cingulate gradients and canonical functional networks, clinical phenotypes, and meta‐analytic behavioral domains were analyzed. A gene expression decoding analysis based on the Allen Human Brain Atlas was performed to advance understanding of how molecular mechanisms relate to hierarchical changes in ESRD.

**Results:**

Across global, network, and regional scales, patients with ESRD showed significant cingulate gradient dysfunction, with these anomalies exhibiting associations across multiple functional domains. Notably, serum urea and hemoglobin levels were correlated with cingulate gradient dysfunction. Spatially, these alterations correlated with genes enriched in neurodegenerative processes. Excitatory and inhibitory neurons' specific transcriptional changes account for most of the observed correlation with ESRD‐specific cingulate gradient alterations.

**Conclusion:**

Our findings highlight ESRD‐related cingulate gradient dysfunction and its links to clinical phenotypes and gene expression profiles, providing critical insights into the neurodegenerative underpinnings of cerebral dysfunction in ESRD.

## Graphical abstract

End‐stage renal disease (ESRD) patients exhibited significant cingulate gradient dysfunction, which was significantly correlated with multiple functional domains, serum urea, and hemoglobin levels. Spatially, these alterations were correlated with genes enriched in neurodegenerative pathways. Excitatory and inhibitory neurons account for ESRD's cingulate gradient dysfunction.

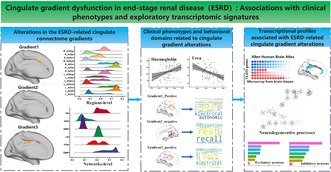

## 1 Introduction

End‐stage renal disease (ESRD), the most advanced stage of chronic kidney disease (CKD), frequently impacts the brain [1, 2] via pathogenic factors including uremic toxin accumulation [3], disordered calcium‐phosphate metabolism [4], and anemia [5]. Patients manifest a broad spectrum of clinical phenotypes, encompassing cognitive decline [1], sensorimotor abnormalities [6], pain [7], fatigue [8], and emotional disturbances [9]. The cingulate cortex, a core limbic structure with extensive cortical–subcortical connectivity [10], subserves emotion regulation, motor coordination, and memory processing [11]. Recent neuroimaging studies [12, 13, 14] have identified the cingulate cortex as one of the most susceptible brain regions in ESRD patients. This vulnerability is primarily manifested as reduced spontaneous neural activity, decreased functional connectivity, impaired neurovascular coupling, and microstructural damage in its subcortical white matter. However, conventional neuroimaging approaches focus on regional activity or pairwise connections and cannot capture the macroscale hierarchical organization of the cingulate connectome. Whether breakdown of this fundamental hierarchical architecture contributes to the multifaceted clinical heterogeneity in ESRD remains unknown.

Hierarchical organization is a fundamental principle of brain structure, function, and gene expression, enabling efficient cross‐domain information integration [15]. Cortical functional gradients represent a novel, principled framework to quantify the continuous, macroscale hierarchical organization of resting‐state functional connectivity (rsFC) [16]. Derived via dimensionality reduction, gradients capture smooth spatial transitions of connectivity patterns and compactly represent core topological features of the connectome [16, 17]. Unlike traditional regional or pairwise metrics, gradient analysis reveals system‐level disruptions of brain hierarchy that underlie cognitive and behavioral dysfunction. The cingulate cortex exhibits three well‐characterized functional gradients [18]. The first principal gradient features a radiating pattern, with transitions from its midregion extending toward both anterior and posterior areas, and is linked to canonical functional networks and their corresponding behavioral domains. The second gradient exhibits an anterior–posterior axis that spans the entire cingulate cortex. Additionally, the third gradient reveals striking differentiation between the subgenual and caudal middle regions and the rest of the cingulate cortex. These gradients provide a unique way to decode cingulate functional heterogeneity. Yet whether ESRD disrupts cingulate functional gradients, how such disruptions relate to clinical phenotypes, and their underlying molecular mechanisms remain completely unexplored. This knowledge gap is clinically critical: ESRD‐related brain dysfunction involves multiple interdependent functional systems (e.g., cognition, emotion, sensation, and motor function) that rely on intact cingulate hierarchy. Traditional local or pairwise measures cannot explain the widespread, coordinated clinical manifestations. Cingulate gradient dysfunction may serve as a unifying neural marker for ESRD‐related cerebral impairment.

Identifying transcriptomic variants associated with diverse clinical phenotypes in patients with ESRD holds significant relevance for advancing etiologic, prognostic, and therapeutic understanding of this critical condition [19]. The functional integrity of the cingulate cortex is tightly linked to its transcriptomic, cellular, and biochemical properties [20]. Unlike large‐sample genome‐wide association studies, transcription‐neuroimaging analyses enable gene discovery in small cohorts [21, 22]. The Allen Human Brain Atlas [23] (AHBA; http://human.brain‐map.org) featuring densely sampled postmortem gene expression data has facilitated identification of genes linked to anatomical connectivity, functional disconnectivity [24], and white matter networks [22]. Additionally, Neurosynth [25] (https://neurosynth.org/) enables automated meta‐analysis of functional MRI data to map neuroimaging‐behavioral associations. Leveraging these tools, we aimed to integrate multimodal data to address key knowledge gaps.

**FIGURE 1:**
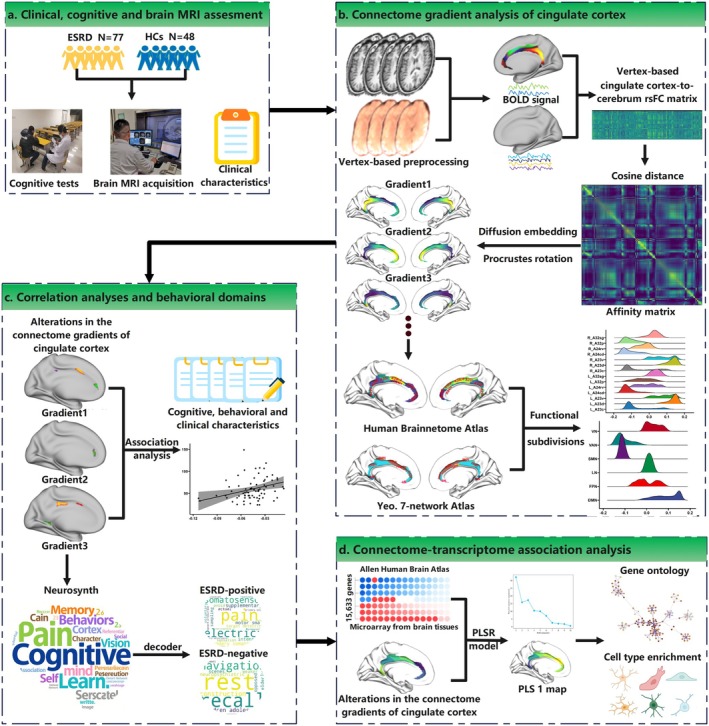
Schematic analysis workflow. (a) Acquire clinical, cognitive, and brain MRI data from 125 participants. (b) Cingulate cortex functional connectivity gradients were derived via diffusion embedding from vertex‐wise rsFC profiles of the cingulate cortex relative to the entire cerebrum. The first three gradients, explaining the largest proportion of connectivity variance, were prioritized. Subsequent analysis explored associations between these cingulate functional gradients and canonical functional networks. (c) Association analyses examined relationships between ESRD‐related alterations in cingulate functional gradients and clinical variables, cognitive performance, and behavioral domains. (d) Connectome‐transcriptome association analysis. Partial least squares (PLS) regression was applied to assess spatial correlations between ESRD‐related cingulate functional gradient alterations and the gene expression matrix. Genes with significant weights in the first PLS component (PLS1) were extracted, and pathway enrichment analyses were conducted to identify relevant biological processes. Finally, PLS1‐associated gene sets were mapped to cell‐type‐specific expression profiles. BOLD, blood oxygen level dependent; ESRD, end‐stage renal disease; HCs, healthy controls; rsFC, resting‐state functional connectivity.

Here, we enrolled 77 ESRD patients and 48 healthy controls (HCs), collecting clinical and resting‐state functional MRI (rs‐fMRI) data to compute vertex‐wise cingulate functional gradients. We then examined associations between gradients and canonical functional networks [26], clinical phenotypes, and meta‐analytic behavioral domains (via Neurosynth [25]). Finally, we linked gradient abnormalities to AHBA transcriptomic data [23] using partial least squares (PLS) regression, followed by functional and cellular enrichment analyses. We hypothesized that: (i) ESRD patients exhibit significant disruptions in cingulate functional gradients compared with HCs; (ii) these gradient alterations correlate with clinical indicators and cognitive/behavioral phenotypes; and (iii) spatial patterns of gradient abnormalities are correlated with normative gene expression profiles from the AHBA, allowing exploratory inference of potential molecular mechanisms. A schematic of the study design is presented in Figure 1.

## 2 Materials and Methods

### 2.1 Participants

As a prospective cohort study, all MRI and clinical data were collected from the same group of participants. We perspectively enrolled 77 right‐handed ESRD patients (52 males, 25 females; mean age: 35 ± 10.24 years) undergoing maintenance hemodialysis between July 2020 and June 2024 (Table 1). The underlying etiologies of ESRD included immunoglobulin A nephropathy (*n* = 32), glomerulonephritis (*n* = 35), and membranous nephropathy (*n* = 10). All ESRD patients were receiving hemodialysis three times weekly, with a shortened midweek interval. Each hemodialysis session lasted approximately 4 h, with a mean urea kinetic modeling (*Kt*/*V*) value of > 1.2 [27]. On the day prior to hemodialysis, all patients completed neuropsychological assessments in a quiet room and underwent blood tests. ESRD patients exclusion criteria: (1) age < 18 or > 60 years; (2) psychiatric or neurodegenerative disorder history; (3) brain lesion history (e.g., stroke, hemorrhage, trauma, tumor, encephalomalacia) per conventional MRI or medical records; (4) current smoking, alcohol abuse, or substance dependence; (5) diabetic nephropathy; (6) visual/auditory impairments (e.g., hearing loss, blurred vision) or other clinically significant symptoms precluding neuropsychological and behavioral domain assessment; (7) claustrophobia or other MRI contraindications. Forty‐eight right‐handed, demographically matched HCs (36 males, 12 females; mean age: 34 ± 8.72 years) were recruited from the local community via advertisements. Inclusion criteria for HCs were age 18–60 years and no history of systemic, neurological, or psychiatric diseases. Notably, gene expression analyses were performed using the public AHBA dataset for spatial correlation, not from direct brain tissue sampling of the present study cohort.

**TABLE 1:** Demographic and clinical characteristics of the participants.

Variable	HC (*n* = 48)	ESRD (*n* = 77)		*t*/*χ* ^2^	*p*
Age (years)	34.63 (8.72)	35.09 (10.24)		0.262	0.794^a^
Sex (M/F)	36 (12)	52 (25)		0.791	0.425^c^
Education (years)	12.29 (2.14)	12.43 (1.90)		0.373	0.710^a^
Dialysis vintage (months)	—	39.60 (28.65)		—	—
Creatinine (μmol/L)	—	920.40 (219.21)		—	—
Urea (mmol/L)	—	23.77 (7.23)		—	—
*Kt*/*V*	—	1.46 (0.21)		—	—
Hemoglobin (g/L)	—	106.82 (20.37)		—	—
Hematocrit (%)	—	32.85 (6.00)		—	—
Cystatin C (μg/mL)	—	6.09 (3.19)		—	—
Potassium (mmol/L)	—	4.81 (0.75)		—	—
Sodium (mmol/L)	—	142.31 (3.28)		—	—
Phosphorus (mmol/L)	—	1.80 (0.51)		—	—
Calcium (mmol/L)	—	2.14 (0.34)		—	—
Parathormone (ng/L)	—	627.78 (456.11)		—	—
**Medication**		***N***	**%**		
AT1‐blocker		67	87	—	—
Beta‐blocker		50	65	—	—
Calcium antagonists		70	91	—	—
Antidepressants		0	0	—	—
Antihistamines		0	0	—	—
Analgesics		0	0	—	—
Vitamin D		55	54	—	—
EPO		75	97	—	—
**MoCA**					
Total score	27.42 (2.22)	23.81 (2.86)		−7.454	< 0.001^a^, ^d^
Visuospatial	4 (1)	4 (2)		−5.463	< 0.001^b^, ^d^
Name	3 (0)	3 (0)		−2.224	0.026^b^, ^d^
Attention	6 (1)	6 (1.5)		−1.722	0.085^b^
Language	3 (1)	2 (0)		−5.329	< 0.001^b^, ^d^
Abstraction	2 (0)	1 (1)		−5.325	< 0.001^b^, ^d^
Orientation	6 (0)	6 (1)		−1.361	0.174^b^
Delayed memory	4 (1)	4 (1)		−5.309	< 0.001^b^, ^d^
**AVLT‐H**					
IR‐S	27.83 (3.69)	25.25 (4.47)		−3.356	0.001^a^, ^d^
SR‐S	10.31 (1.20)	9.57 (1.66)		−2.684	0.008^a^, ^d^
LR‐S	10.25 (1.28)	9.00 (1.74)		−4.302	< 0.001^a^, ^d^
REC‐S	11.83 (0.48)	11.32 (1.01)		−3.278	0.001^a^, ^d^
TMT	41.92 (13.33)	60.55 (28.35)		4.264	< 0.001^b^, ^d^
PSQI	5 (3.5)	9.50 (6)		5.136	< 0.001^b^, ^d^
PHQ‐9	—	7.50 (6.5)		—	—
SSLS	28 (0.98)	41.5 (16)		−6.511	< 0.001^b^, ^d^
FIS‐20	19 (17.5)	27 (18.75)		−2.167	0.030^b^, ^d^
HIT‐9	36 (2)	46 (14.5)		4.150	< 0.001^b^, ^d^
BDI	7.92 (5.23)	16.09 (10.21)		5.135	< 0.001^a^, ^d^
BAI	25.17 (2.80)	28.65 (6.06)		3.736	< 0.001^a^, ^d^

*Note:* Unless otherwise indicated, data are mean (standard deviation).

Abbreviations: AVLT‐H, auditory verbal learning test–Huashan version; BAI, Beck Anxiety Inventory; BDI, Beck Depression Inventory; ESRD, end‐stage renal disease; FIS‐20, Fatigue Impact Scale‐20; HCs, health controls; HIT‐6, Headache Impact Test‐6; IR‐S, immediate recall score; LR‐S, long‐term recall score; MoCA, Montreal cognitive assessment; PHQ‐9, Patient Health Questionnaire‐9; PSQI, Pittsburgh Sleep Quality Index; REC‐S, recognition score; SR‐S, short‐term recall score; SSLS, Somatic Symptom Likert Scale; TMT, trail‐making test.

^a^ Analyzed with the independent two‐sample *t*‐test; data in parentheses have a 95% confidence interval. Data are mean (standard deviation).

^b^ Analyzed with the Mann–Whitney *U*‐test, data are median (range interquartile).

^c^ Analyzed with the chi‐square test.

^d^ Statistically significant difference after controlling for age, sex, and education level.

### 2.2 Neuropsychological and Behavioral Domain Assessment

Behavioral domain measures comprised the Pittsburgh sleep quality index (PSQI), patient health questionnaire‐9 (PHQ‐9), somatic symptom Likert scale (SSLS), fatigue impact scale‐20 (FIS‐20), and headache impact test‐6 (HIT‐6). Neuropsychological assessments included the auditory verbal learning test‐Huashan version (AVLT‐H), Montreal cognitive assessment (MoCA), trail‐making test (TMT), Beck depression inventory (BDI), and Beck anxiety inventory (BAI).

### 2.3 MRI Data Acquisition and Preprocessing

All MRI data were collected for each participant using a 3.0 T Discovery MR750 scanner equipped with an eight‐channel phased‐array head coil. MRI protocols included T2‐weighted periodically rotated overlapping parallel lines with enhanced reconstruction (T2‐PROPELLER) images, individual high‐resolution T1‐weighted structural images, and rs‐fMRI scans for all participants. Participants were instructed to lie quietly with eyes closed and stay awake during scanning. Foam padding and earplugs were used to minimize head motion and scanner noise. Scanning was discontinued if participants reported discomfort or were unable to complete the procedure. Following scanning, all participants confirmed their cooperation. T2‐PROPELLER images with parameters: echo time (TE)/repetition time (TR) = 84/9638 ms, matrix = 256 × 256, refocus angle = 142°, slice thickness = 6 mm, slice gap = 0.6 mm, were acquired to exclude intracranial lesions. T1‐weighted anatomical data were obtained using a three‐dimensional brain volume imaging sequence (3D‐BRAVO) with parameters: TE/TR = 3.2/8.2 ms, flip angle (FA) = 15°, matrix = 256 × 256, slice thickness = 1 mm, slice gap = 0 mm, and number of slices = 140. T1‐weighted anatomical images were acquired prior to the rs‐fMRI protocol to allow participants to acclimate to the MR environment. The rs‐fMRI data were collected using an echo‐planar imaging (EPI) sequence with the following parameters: TE/TR = 50/2000 ms, matrix = 64 × 64, field‐of‐view (FOV) = 240 × 240 mm^2^, FA = 90°, slice thickness = 4 mm, slice gap = 0 mm, number of slices = 45. Each rs‐fMRI acquisition lasted 6 min and 10 s, comprising 185 functional volumes. After the rs‐fMRI scan, all participants were asked to confirm that they remained awake throughout the scanning process. Preprocessing of anatomical and functional data was performed using fMRIPrep v23.1.4 [28] and the eXtensible Connectivity Pipeline [29, 30], see Supporting Information.

### 2.4 Cingulate Functional Gradient Analysis

To generate connectome gradients for each participant, we used the open‐source tool BrainSpace (https://github.com/MICA‐MNI/BrainSpace) [17]. First, the Human Brainnetome Atlas [31]—built via connectivity‐based parcellation—was used to parcellate the cingulate cortex into 2685 vertices. This atlas was validated using rsFC, tractography‐based anatomical connectivity, and meta‐analytic functional‐behavioral decoding, with initial parcellation derived from diffusion MRI probabilistic tractography of connectional architecture. Second, preprocessed blood oxygen level‐dependent (BOLD) images were *z*‐score standardized and concatenated across subjects to generate group‐level BOLD time courses. Third, using these group time courses, we created a vertex‐wise cingulate‐to‐cerebrum rsFC matrix (2685 × 56,727) via Pearson correlations between time courses of individual cingulate vertices and all cerebrum vertices (excluding the cingulate). The matrix underwent Fisher's Z‐transformation to enhance normality, then was thresholded to retain only the top 10% of connections per row (other values set to zero). Fourth, we used cosine distance to compute a positive, symmetric affinity matrix reflecting connectivity profile similarity between cingulate vertex pairs. We then applied diffusion map embedding [16, 32]—a noise‐robust, computationally efficient nonlinear dimensionality reduction method—to extract principal gradient components, which explain functional connectome variance in descending order. This method converts connectivity relationships into distances in a high‐dimensional space, stabilizing pattern representation: regions with similar connectivity cluster closely, while sparsely connected regions are farther apart. Following prior recommendations [16, 32], we set the manifold learning parameter *α* = 0.5 during embedding to preserve global relationships. Resultant gradient maps were aligned across individuals via Procrustes rotation [33]. For each gradient map, three global gradient metrics were calculated [17]: range, variance, and explained ratio. Specifically, the gradient range quantifies the difference between the maximum positive and negative values across brain regions for a given gradient. The gradient variance represents the standard deviation of gradient scores across the brain. The explained ratio denotes the percentage of connectivity variance accounted for by a given gradient. We primarily focused on ESRD‐related alterations in the first three gradients.

### 2.5 Cingulate Gradient Subregions Analysis and Functional Networks Analysis

The Human Brainnetome Atlas [31] can divide the cingulate cortex into seven subregions in each of the left and right hemispheres: dorsal area 23 (A23d), rostroventral area 24 (A24rv), pregenual area 32 (A32p), ventral area 23 (A23v), caudodorsal area 24 (A24cd), caudal area 23 (A23c), and subgenual area 32 (A32sg). We extracted all vertex gradient values for each of these 14 cingulate subdivisions separately and calculated the mean gradient value for each subdivision. A cingulate functional atlas was first created using a custom parcellation approach [18]: specifically, we computed Pearson's correlation coefficients between the BOLD time course of each cingulate vertex and the average BOLD time course of each functional network. Each vertex was then assigned to the functional network with the highest correlation coefficient. This process was repeated for all cingulate vertices, yielding a cingulate functional atlas with seven subdivisions, each corresponding to one of the seven canonical networks. Finally, we extracted gradient values for vertices within these seven functional networks and calculated the mean gradient value for each network.

### 2.6 Statistical Analyses

The between‐group differences in demographics (age and educational level), neuropsychological variables, and behavioral domain measures were examined using the Kolmogorov–Smirnov test, Levene's test, and independent two‐sample *t*‐tests, with analyses conducted in Statistical Package for the Social Sciences (SPSS Statistics, version 22.0; IBM, Armonk, NY, USA). Specifically, the Kolmogorov–Smirnov test assessed the normality of data distribution, Levene's test examined variance homogeneity, and Student's *t*‐test evaluated differences in means. A chi‐square test was applied to compare group differences in sex distribution. Multiple linear regression analysis was employed to adjust for the effects of age, sex, educational level, and mood disorder on group differences in neuropsychological scores.

To assess between‐group differences in cingulate functional gradients, a general linear model incorporating age, sex, and education level as covariates was employed. For global gradient metrics (range, variance, and explained ratio), statistical significance was set at *p* < 0.05 following false discovery rate (FDR) correction. For vertex‐based gradient score maps, we used PALM (https://web.mit.edu/fsl_v5.0.10/fsl/doc/wiki/PALM.html) to compute threshold‐free cluster enhancement (TFCE) and adjusted it to a threshold of *p* < 0.05 family‐wise error (FWE). For cingulate subregion and functional network gradient metrics, statistical significance was set at *p* < 0.05 following FDR correction. Pearson or Spearman correlation analyses were performed to assess relationships between gradient alterations and clinical characteristics, neuropsychological variables, and behavioral domain measures, respectively. The significance threshold was set at *p* < 0.05 following FWE correction.

### 2.7 Meta‐Analytic Behavioral Domains Related to Cingulate Gradient Alterations

To investigate the behavioral relevance of the first three connectome gradients in patients with ESRD, we examined their associations with meta‐analytic behavioral terms derived from NeuroSynth [25] (https://neurosynth.org/). The NeuroSynth database curates activation maps (*z*‐statistics) for a broad spectrum of behavioral terms, each capturing conceptually distinct dimensions of human behavior. First, we partitioned ESRD‐related alterations across each cingulate functional gradient (based on the Human Brainnetome Atlas [31]) into two contrast maps: ESRD‐positive maps (i.e., gradient values significantly higher in ESRD patients relative to HCs) and ESRD‐negative maps (i.e., gradient values significantly lower in ESRD patients relative to HCs). Second, to link these gradient alterations to behavioral functions, we applied the “decoder” function in NeuroSynth to compute spatial correlations between the ESRD‐positive/negative maps and the meta‐analytic activation map of each behavioral term in the NeuroSynth database (version 7). Following this, we selected the top 30 behavioral terms with the strongest correlations and further validated these associations via permutation tests (10,000 iterations) with FDR correction. Spatial autocorrelations in the imaging data were accounted for via generative modeling to ensure robust estimation of significance for each behavioral term's correlation coefficient.

### 2.8 Transcriptional Profiles Related to Cingulate Gradient Alterations

Following the established pipeline for connectome‐transcriptome analysis [34], the AHBA dataset was processed using the abagen toolbox [35](https://github.com/rmarkello/abagen) in conjunction with the Human Brainnetome Atlas [31], focusing on the cingulate cortex. Notably, this transcriptomic analysis serves to infer potential molecular correlates of imaging abnormalities, rather than providing direct evidence of disease‐specific genomic or transcriptomic alterations in the present ESRD patient cohort. These normalized expression data were acquired from postmortem brain samples of six neurotypical adult donors (Table S5). Main steps included probe‐to‐gene reannotation, data filtering, probe selection, sample assignment, gene filtering, and correction for interindividual and intersample variability. First, microarray probes were reannotated using data from the practical guide for gene expression data preprocessing, and probes without a valid Entrez ID match were discarded. Next, probes were filtered by expression intensity relative to background noise: those with intensity below background in ≥ 50% of samples across all donors were excluded. For genes indexed by multiple probes, we selected the probe with the most consistent regional variation pattern across donors. The MNI coordinates of tissue samples were updated to those from nonlinear registration with ANTs software. Samples were assigned to regions in the cingulate cortex if their MNI coordinates lay within 2 mm of a given parcel. To minimize misassignment, sample‐to‐region matching was constrained by hemisphere and gross structural divisions. All tissue samples unassigned to any atlas region were discarded. Intersubject variation was mitigated by normalizing tissue sample expression values across genes using a robust sigmoid function. Gene expression values were then normalized across tissue samples via the same procedure. Samples assigned to the same region were first averaged per donor, then aggregated across donors. Finally, for each of the 14 subregions of the cingulate cortex (as defined by the Human Brainnetome Atlas [31]), gene expression levels were estimated by averaging values across all samples from the six donors. This process yielded a gene expression matrix (14 regions × 15,633 genes). Specifically, the *t*‐statistic map of between‐group cingulate gradient alterations—defined according to the Human Brainnetome Atlas—served as the response variable, with the normalized gene expression matrix as the predictor variable. The fitted PLS regression model generated multiple components, each representing a linear combination of predictor variables that explained variance in the response variable. We retained the first component (PLS1) of the model, as it typically captures the largest proportion of variance in the response variable. The significance of the variance explained by PLS1 was evaluated via permutation tests (10,000 times) incorporating spatial autocorrelation correction. Additionally, we used a bootstrapping approach to assess and correct for estimation errors in quantifying each gene's contribution weight to PLS1. Corrected weights were then computed as the ratio of raw weights to the estimated bootstrapped error. Based on these corrected weights, we ranked the genes and derived gene lists with significantly positive and negative weights, denoted as PLS1+ and PLS1−, respectively.

Metascape [36] offers a one‐click analysis interface for transcriptomic gene lists, with an automated workflow comprising four key components: identifier conversion, gene annotation, membership search, and enrichment analysis. In our enrichment analysis, the input gene list was compared against thousands of gene sets annotated to specific biological processes, protein localizations, enzymatic functions, pathways, or other features. Metascape uses the hypergeometric test and Benjamini–Hochberg *p* value correction to identify ontology terms with more overlapping genes with the input list than expected by chance, reporting these overrepresented gene sets as putative biological insights. For post‐processing, Kappa similarities between all enriched terms were calculated, and a 0.3 threshold was used to hierarchically cluster terms into groups of similar terms. This clustering reduces redundancy by grouping similar terms, avoiding interpretive confusion from redundant ontology reporting. We conducted enrichment analysis to characterize the biological pathways associated with ESRD. The PLS1 ± gene lists were analyzed separately using GO biological processes, Reactome gene sets, KEGG pathways, WikiPathways, Canonical Pathways, and CORUM within Metascape [36]. Identified enrichments were filtered using a significance threshold of FDR‐corrected *p* < 0.05. In order to map PLS1 genes to specific cell types, we intersected the PLS1 gene list with the respective gene set of each cell type. The respective gene set for each cell type was derived from prior studies [22, 37]. These cell types were classified into seven categories [38]: astrocytes, endothelial cells, microglia, oligodendrocytes, oligodendrocyte precursor cells, excitatory neurons, and inhibitory neurons. For each cell type, the *p* value corresponding to the number of overlapping genes was determined via permutation test (10,000 times), followed by FDR correction, with a significance threshold of *p* < 0.05.

## 3 Results

### 3.1 Demographic and Clinical Phenotypic Results

Table 1 presents the demographic and clinical characteristics between groups. No significant between‐group differences were observed in age, sex, or educational level. Regarding neuropsychological assessments, ESRD patients performed significantly worse than HCs on multiple measures: subscales of the AVLT‐H, including immediate recall (*p* = 0.001), short‐term recall (*p* = 0.008), long‐term recall (*p* < 0.001), and recognition (*p* = 0.001); subdomains of the MoCA, such as total score (*p* < 0.001), visuospatial function (*p* < 0.001), naming (*p* = 0.026), language (*p* < 0.001), abstraction (*p* < 0.001), and delayed memory (*p* < 0.001); as well as the TMT (*p* < 0.001), BAI (*p* < 0.001), and BDI (*p* < 0.001). Additionally, compared with HCs, ESRD patients exhibited poorer sleep quality (PSQI, *p* < 0.001), more severe somatic symptoms (SSLS, *p* < 0.001), increased fatigue (FIS‐20, *p* = 0.03), and more frequent headache (HIT‐6, *p* < 0.001).

### 3.2 Alterations in the Cingulate Functional Gradients in ESRD Patients

**FIGURE 2:**
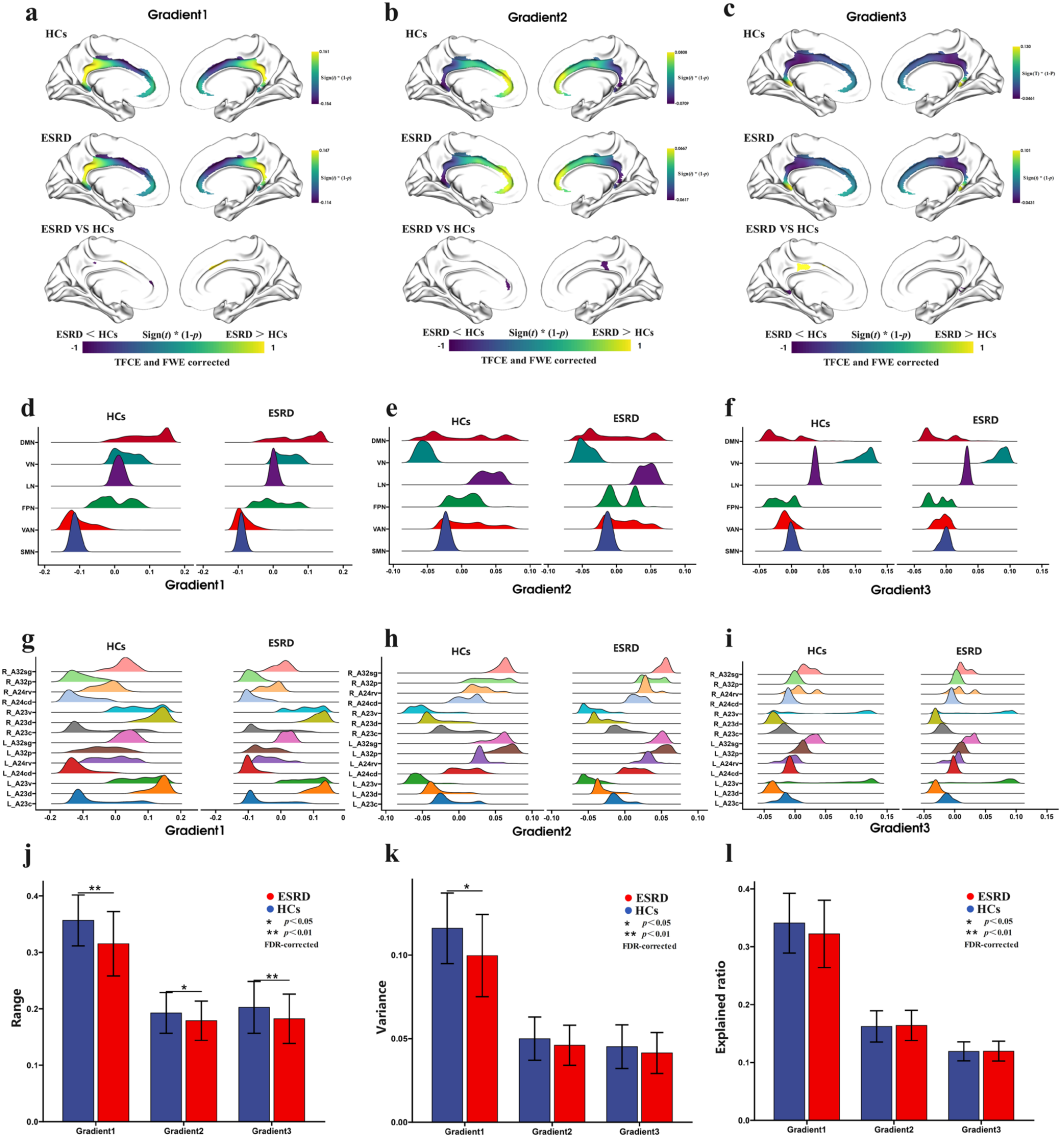
Connectome gradient mapping of the cingulate cortex. (a) Gradient 1 exhibited a radiating topographic organization, transitioning from the midcingulate cortex toward anterior and posterior sectors. (b) Gradient 2 followed an anteroposterior axis spanning the cingulate cortex. (c) Gradient 3 revealed pronounced differentiation of subgenual and caudomedial cingulate regions from other cortical sectors. (a–c) Vertex‐wise coloring reflects connectivity similarity, with conserved gradient topographies across groups. ESRD‐related decreases/increases in gradient values are encoded as cold/warm hues. Statistical significance was defined by *p* < 0.05 after TFCE and FWE correction. (d–f) Group comparisons of gradient scores using the Yeo seven‐network parcellation. (g–i) Group difference analysis of gradient scores via the Human Brainnetome Atlas parcellation. (j–l) Group differences in global metric properties of the first three gradients. A23c, caudal area 23; A23d, dorsal area 23; A23v, ventral area 23; A24cd, caudodorsal area 24; A24rv, rostroventral area 24; A32p, pregenual area 32; A32sg, subgenual area 32; DMN, default mode network; FPN, frontoparietal network; FWE, family‐wise error; LN, limbic network; SMN, sensorimotor network; TFCE, threshold‐free cluster enhancement; VAN, ventral attention network; VN, visual network.

We focused on ESRD‐related alterations in the top three gradients, which together explained 61% of the total variance (Figure S1). The distribution patterns of gradients 1–3 were consistent with previous findings on cingulate gradients in healthy adults [18] (Figure 2a–f). At the vertex level, group comparisons for gradient 1 showed decreased values in ESRD patients within L_A23d and L_A32sg, alongside increased values predominantly in L_A24cd, R_A23c, and R_A24cd (TFCE, *p* < 0.05 FWE corrected; Figure 2a, Table S1). At the Human Brainnetome Atlas [31] level, ESRD patients had lower gradient scores in L_A24rv and R_A32sg but higher scores in L_A24cd and R_A24cd relative to HCs (FDR corrected; Figure 2g, Table S1). At the network level, ESRD patients demonstrated reduced gradient scores in the DMN but elevated scores in the VAN compared to HCs (FDR corrected; Figure 2d, Table S1). Group‐level statistical comparisons of global gradient metrics (Figure 2j–l, Table S2) further demonstrated that ESRD patients exhibited a narrower range across all three gradients (gradient 1: *p* < 0.01; gradient 2: *p* < 0.05; gradient 3: *p* < 0.01; all FDR corrected), as well as diminished spatial variation compared to HCs (gradient 1: *p* < 0.05, FDR corrected).

### 3.3 Clinical Phenotypes Associated With Cingulate Gradient Alterations

**FIGURE 3:**
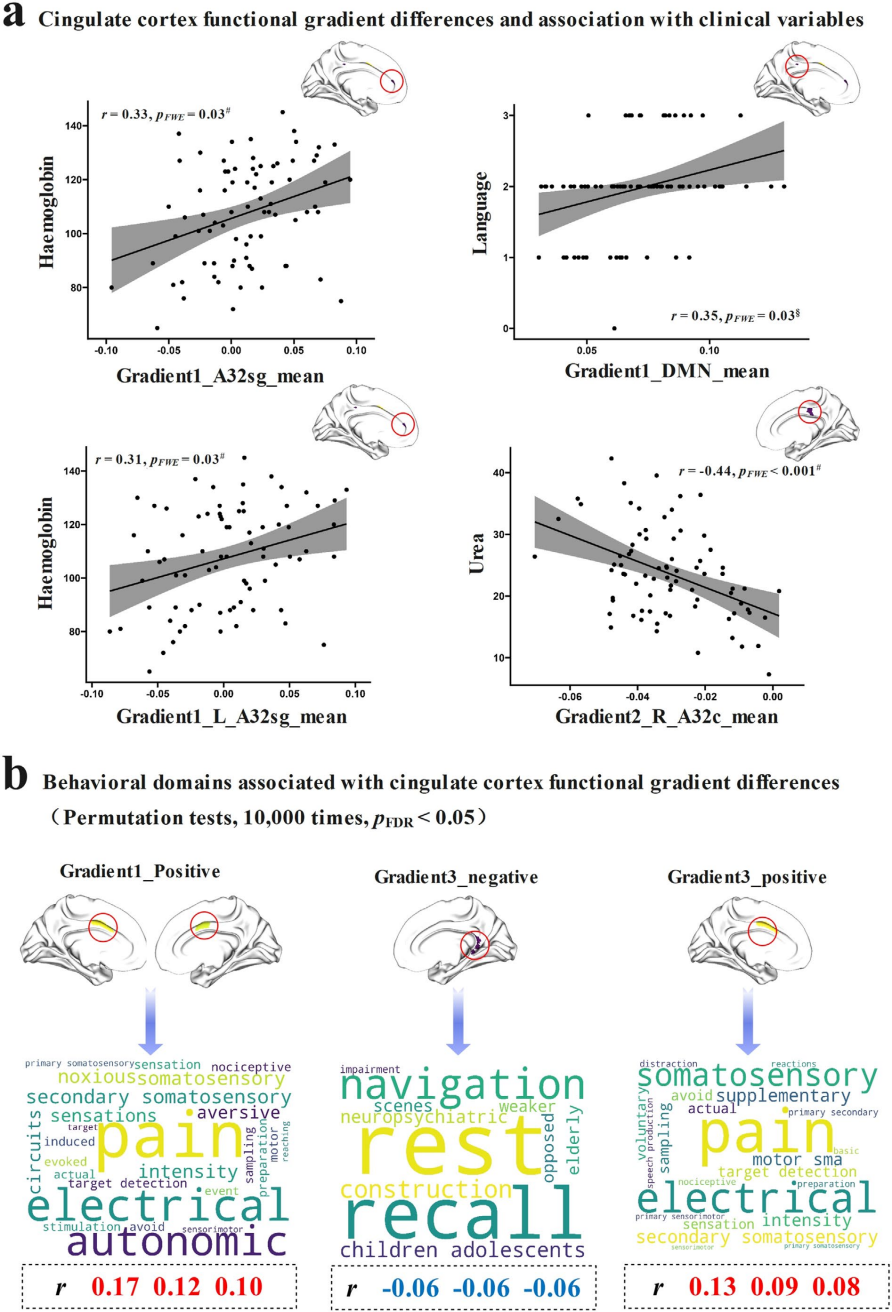
Association analysis of ESRD‐related cingulate functional gradient alterations with clinical, neuropsychological, and behavioral domains. (a) Pearson (#) or Spearman (§) correlation analyses were used to assess relationships between gradient metrics alterations and clinical and cognitive variables across all patients (*p* < 0.05, FWE corrected). (b) Behavioral domains linked to cingulate functional gradient differences. Depicted are word clouds illustrating behavioral domains associated with brain regions exhibiting lower (cold) or higher (warm) between‐group gradient metrics. Font size of each behavioral domain is proportional to the correlation coefficient (*r*) between the between‐group Z‐map of the first three gradients and the Neurosynth‐generated meta‐analytic map for each behavioral term. All correlations reached significance (*p*_perm_ < 0.05, FDR corrected).

Correlation analyses between gradient differences and clinical phenotypes revealed that in ESRD patients, a positive association was observed between gradient 1's A32sg and hemoglobin levels (Pearson correlation: *r* = 0.33, *p*_FWE_ = 0.03), with a further positive association between gradient 1's L_A32sg and hemoglobin levels (Pearson correlation: *r* = 0.31, *p*_FWE_ = 0.03). Additionally, a negative association was found between gradient 2's R_A32c and serum urea levels (Pearson correlation: *r* = −0.44, *p*_FWE_ < 0.001). Separately, correlation analyses between gradient differences and neuropsychological scores showed that gradient 1's DMN was positively correlated with language scores in ESRD patients (Spearman correlation: *r* = 0.35, *p*_FWE_ = 0.03) (Figure 3a).

### 3.4 Meta‐Analytic Behavioral Domains Related to Cingulate Gradient Alterations

Figure 3b and Tables S3 and S4 show the results of spatial correlation between ESRD‐related gradient alterations and the meta‐analytic map of behavioral terms (Permutation tests, 10,000 times, *p*_FDR_ < 0.05). The regions with higher scores of gradient 1 in ESRD patients were correlated with several meta‐analytic behavioral terms mainly involved in motor and sensory processes such as pain, autonomic, motor, somatosensory, and secondary somatosensory. The regions with higher scores of gradient 3 in ESRD patients were correlated with pain, motor, somatosensory, sensorimotor, autonomic, and sensation. The regions with lower gradient 3 scores in ESRD patients were correlated with several high‐order cognitive terms, including recall, navigation, and construction.

### 3.5 Transcriptional Profiles Related to Cingulate Gradient Alterations

**FIGURE 4:**
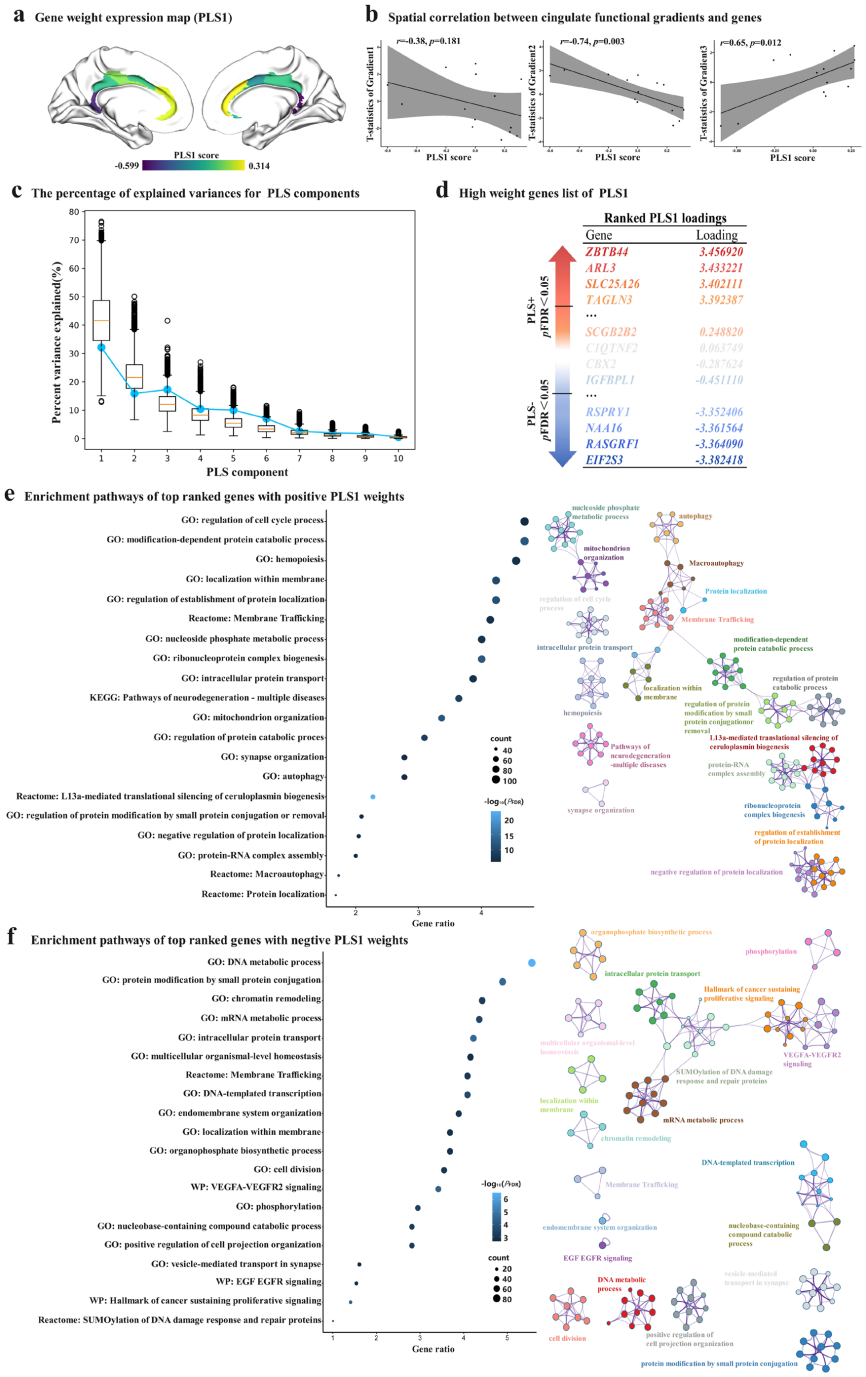
Transcriptional profiles associated with ESRD‐related cingulate functional gradient alterations. (a) Brain map of the first component of partial least squares (PLS1) scores. (b) Correlation between the PLS1 score and functional gradient changes in ESRD patients. Each dot represents a region. (c) The percentage of explained variance for PLS components. (d) Ranked list of PLS1‐positive (PLS1+) and PLS1‐negative (PLS1−) genes (*p* < 0.05, FDR corrected). (e, f) Functional enrichment analysis of top ranked genes with PLS1+ (e) and PLS1− (f) weights. The bubble diagram illustrate the number of genes overlapping with each annotation, where bubble color indicates significance level and bubble size corresponds to the number of genes in each GO term or pathway. Metascape‐derived enrichment network diagrams depict inter‐ and intra‐cluster similarities among enriched terms/pathways for PLS1+ (e) and PLS1− (f) genes. Nodes represent individual terms or pathways (node size reflects the number of genes in each term/pathway), while edges denote interterm connections; node colors distinguish clusters. GO, Gene Ontology; KEGG, Kyoto Encyclopedia of Genes and Genomes; WP, WikiPathways.

PLS1 explained 32.1% of the variance in the ESRD‐related cingulate functional gradient alterations (*p*_spin_ = 0.009) (Figure 4a,c). We found that the PLS1 score map was spatially correlated with the *t* statistics maps of gradient 2 (*r* = −0.74, *p* = 0.003) and gradient 3 (*r* = 0.65, *p* = 0.012) (Figure 4b). After ranking the 15,633 genes in terms of corrected weights, we identified 2231 genes with significant positive PLS1 weights (PLS1+ gene list, FDR‐corrected *p* < 0.05) and 479 genes with significant negative PLS1 weights (PLS1− gene list, FDR‐corrected *p* < 0.05) (Figure 4d). These significant spatial correlations between PLS1 scores and *t* statistics suggested that positively or negatively weighted genes were overexpressed in brain regions where gradient properties were decreased or increased, respectively, in the ESRD patients compared with the HCs. Specifically, PLS1+ genes were significantly enriched in pathways involved in cell cycle regulation, protein metabolism, and neurodegenerative processes (*p* < 0.05, FDR‐corrected; Figure 4e and Table S6). In contrast, PLS1− genes were enriched in pathways related to DNA and mRNA metabolism processes, protein modification, as well as cellular proliferation and signaling processes (Figure 4f and Table S7).

**FIGURE 5:**
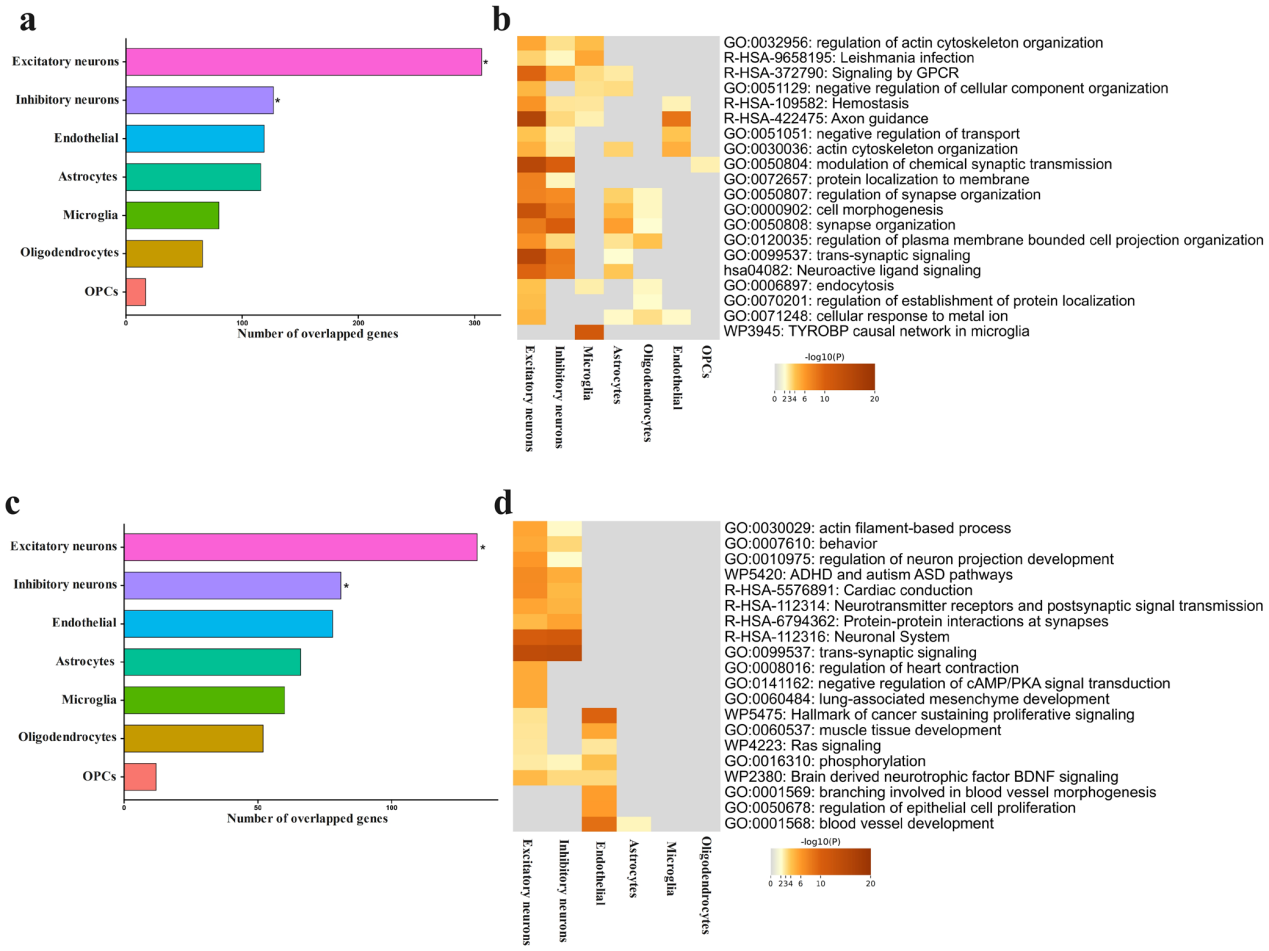
Cell type‐specific expression to changes in cingulate functional gradient‐related genes. Enrichment pathways of cell‐specific highly‐ranked genes with PLS1+ (a, b) and PLS1− (c, d) weights. Left panel (a, c) is the number of highly‐ranked genes in each cell type. Right panel (b, d) is the enrichment analysis in each cell type. An asterisk denotes FDR corrected *p*_perm_ < 0.05. A key of heatmap indicates the statistically enriched terms in each cell type.

According to the gene distribution from seven canonical cell types [38], several PLS1+ genes were significantly related to excitatory neurons (*n* = 306, adjusted *p*_perm_ = 0.0006, FDR‐corrected) and inhibitory neurons (*n* = 127, adjusted *p*_perm_ = 0.0007, FDR‐corrected) (Figure 5a); while several PLS1− genes were also significantly related to excitatory neurons (*n* = 132, adjusted *p*_perm_ = 0.002, FDR‐corrected) and inhibitory neurons (*n* = 81, adjusted *p*_perm_ = 0.032, FDR‐corrected) (Figure 5c). Enrichment analysis of cell type‐specific genes (both PLS1+ and PLS1−) revealed that changes in the cingulate gradient among individuals with ESRD were significantly enriched in biological processes associated with excitatory and inhibitory neurons. Specifically, cingulate gradient changes identified in neuronal cells (based on PLS1+ genes) were primarily enriched in synapse organization, modulation of chemical synaptic transmission, trans‐synaptic signaling, and axon guidance (Figure 5b). In contrast, those identified in neuronal cells (based on PLS1− genes) were mainly enriched in trans‐synaptic signaling and neuronal system pathways (Figure 5d).

## 4 Discussion

Our study presents the first integrated investigation into cingulate functional gradient alterations, clinical phenotypes, and transcriptomic signatures in patients with ESRD. We comprehensively analyzed cingulate gradient abnormalities, clinical phenotypes, and transcriptional expression patterns. Across global, network, and regional scales, we identified marked cingulate gradient disruptions in ESRD patients, and these alterations were linked to multiple functional domains. Notably, serum urea and hemoglobin levels were significantly correlated with cingulate gradient dysfunction in this cohort. Spatially, these ESRD‐associated gradient alterations were correlated with the expression of genes enriched for pathways mainly involved in neurodegenerative processes. Furthermore, cell type annotation revealed that ESRD‐associated transcriptional signatures were predominantly implicated in biological processes specific to excitatory and inhibitory neurons.

### 4.1 ESRD‐Related Cingulate Gradients Dysfunction

Cortical functional hierarchy is a fundamental principle of human brain organization. Functional gradients govern cortical information transmission and support the progressive integration of sensory inputs, which further transforms into abstract cognitive and behavioral representations [39]. At the global level, principal cingulate connectome gradient mapping showed that both groups presented a radiating axis extending from the midcingulate toward the anterior and posterior cingulate cortex. However, ESRD patients displayed a narrower gradient range and reduced spatial variance, indicating less differentiated interregional connectivity. Maximal divergence in connectivity dissimilarity and physical distance sustains the complete information processing cascade. This cascade spans from concrete stimulus encoding to abstract conceptual integration and strengthens interregional differentiation [40]. Such organizational characteristics facilitate high‐order abstract cognition and reduce noise interference during neural information input [40].

Previous ESRD neuroimaging studies mainly focused on regional neural activity, pairwise functional connectivity, or local network abnormalities [12, 41, 42, 43, 44]. These methods are limited to local or discrete connections and cannot reflect the macroscale hierarchical organization of the cingulate cortex. In contrast, cingulate functional gradient analysis quantifies continuous, system‐level changes in connectivity architecture, providing a more comprehensive and mechanistically interpretable marker of brain dysfunction in ESRD patients. In network‐level analyses, ESRD patients showed decreased gradient scores within the DMN and VN, alongside elevated scores in the VAN compared with HCs. These findings indicate reduced gradient values in cingulate subregions affiliated with the DMN and VN, and increased values in VAN‐related subdivisions. Multiple cingulate subregions serve as core hubs for coordinating large‐scale brain network interactions [10]. In the present study, cingulate‐associated functional networks showed evident structural homogenization, accompanied by disrupted connectivity between the cingulate cortex and core large‐scale networks. Meanwhile, the hierarchical distinction across cingulate functional subregions became progressively diminished. Such hierarchical breakdown may serve as a core neural mechanism responsible for widespread cognitive and behavioral dysfunction in ESRD patients.

### 4.2 Cingulate Gradient Dysfunction and Behavioral Domains in ESRD Patients

The diverse clinical phenotypes of patients with ESRD underlie the complexity of brain imaging manifestations. A core value of human neuroimaging lies in brain decoding. It infers individual behavioral characteristics based on measurable brain features. In this study, we explored the relationships of cingulate functional gradient abnormalities with clinical indicators and cognitive–behavioral scales in ESRD. Relative to HCs, ESRD patients showed obvious cognitive deficits, emotional disturbances including anxiety and depression, sleep disruption, severe somatic symptoms, persistent fatigue, and frequent headaches. In ESRD patients, gradient 1 values of the posterior cingulate cortex within the DMN were negatively correlated with language performance. As a key hub of the DMN, the posterior cingulate gyrus participates in the dynamic regulation of fine semantic discrimination, linguistic parsing and high‐order semantic processing [45, 46]. Clinically, ESRD is commonly accompanied by motor dysfunction, sensory deficits, and advanced cognitive deficits [47, 48, 49, 50]. Notably, no significant correlations were detected between cingulate gradient alterations and somatic symptoms, fatigue or headaches. A reasonable explanation is that ESRD‐related cingulate gradient changes may not be sensitive enough to reflect these somatic symptoms. Moreover, behavioral decoding was performed using the Neurosynth database [25]. Multiple behavioral domains were mapped to brain regions with ESRD‐related cingulate gradient dysfunction. Specifically, cingulate subregions with elevated gradient scores in ESRD patients were predominantly linked to motor and sensory processing, whereas regions with reduced gradient values were associated with high‐order cognitive functions. Collectively, our connectome‐based functional gradient findings provide novel neural evidence for the mechanistic basis of cognitive and behavioral abnormalities in ESRD.

### 4.3 Cingulate Gradient Dysfunction and Risk Factors in ESRD Patients

We found that elevated serum urea and decreased hemoglobin levels were closely associated with altered cingulate functional gradients in ESRD patients. These findings suggest that uremic toxins and anemia may critically contribute to cingulate gradient abnormalities in this population. High serum urea is extremely prevalent among individuals with ESRD. As a major end product of protein metabolism, urea participates in urine concentration and water balance regulation and also serves as a reliable biomarker of disease severity and dialysis dependence. Excess urea not only drives systemic uremic syndrome but also exerts direct and indirect chronic neurotoxic effects [51, 52, 53]. Accumulating preclinical evidence [54, 55] has confirmed that urea toxicity triggers pro‐inflammatory signaling and enhances oxidative stress. These pathological processes upregulate proapoptotic gene expression, accelerate cellular senescence, and impair synaptic plasticity. Anemia is a well‐established risk factor for cerebral dysfunction across CKD stages, especially in patients with ESRD [56]. ESRD leads to impaired erythropoietin production, disturbed iron metabolism, and shortened red blood cell survival. Together, these changes reduce blood oxygen‐carrying capacity, induce cerebral hypoxia, and disturb brain energy metabolism [56]. Cerebral gray matter is highly vulnerable to hypoxic injury. Persistent oxygen insufficiency disrupts neuronal energy homeostasis, impairs neural signal transmission, and disturbs the synthesis and release of key neurotransmitters such as acetylcholine and dopamine. Such pathological changes further aggravate progressive cerebral dysfunction [57]. In summary, our results indicate that dysfunctional cingulate gradients in patients with ESRD may be adversely impacted by urea accumulation and anemia, suggesting that clinical practice should prioritize the correction of anemia and the timely clearance of urea.

Human imaging genetics has recently emerged as a powerful methodological framework for unraveling the molecular basis of brain connectome organization [38]. Using the PLS regression method, we first identified cortical patterns of weighted gene expression that colocalized significantly with cingulate functional gradient alterations in ESRD. We further delineated key biological processes and cellular characteristics potentially contributing to cingulate gradient dysfunction in this disease. In summary, our findings indicate that biological processes associated with ESRD‐related cingulate gradient changes—including cell cycle regulation, protein metabolism, and neurodegenerative pathways—may be upregulated. In contrast, processes such as DNA and mRNA metabolism, protein modification, cellular proliferation, and signal transduction may be downregulated. These core biological processes, namely aberrant proteostasis, disrupted energy metabolism, DNA/mRNA defects, and synaptic/neuronal network dysfunction, are well‐recognized hallmarks of neurodegenerative diseases [58]. Notably, nephrologists and neuroscientists have long emphasized the neurodegenerative hypothesis of brain injury in CKD [1]. This hypothesis proposes that impaired renal function in CKD triggers a cascade of pathophysiological changes, including accumulation of uremic neurotoxins, chronic inflammation, and damage to the neurovascular unit. These alterations directly or indirectly disrupt brain energy metabolism, proteostasis, and synaptic/neuronal network integrity, thereby initiating neurodegenerative processes and manifesting as brain injury (e.g., cognitive deficits, sensorimotor dysfunction) [1, 3]. Thus, we speculate that the molecular mechanisms underlying cingulate functional gradient dysregulation in ESRD may align with those of neurodegenerative diseases. This observation provides further support for the neurodegenerative hypothesis of brain injury in ESRD patients.

Interestingly, cell type–specific expression analyses for both PLS+ and PLS− showed that, among genes linked to cingulate functional gradient dysregulation in ESRD patients, overlapping genes related to excitatory and inhibitory neurons were significantly more abundant than those of other cell types. These genes were mainly enriched in biological processes such as synapse organization, modulation of chemical synaptic transmission, trans‐synaptic signaling, and axon guidance. The balance between excitatory and inhibitory neuronal activity is essential for maintaining normal cognitive function. Disruption of this equilibrium acts as a critical driver of neurodegeneration and large‐scale brain network dysfunction [59], and it is closely implicated in cognitive impairment in Alzheimer's disease [60]. Accumulating evidence indicates that guanidine‐containing uremic toxins impair hippocampal synaptic transmission in CKD rodents and disturb central excitatory‐inhibitory balance. This highlights that CKD‐related neurodegenerative brain changes substantially contribute to the onset and progression of cognitive deficits [61]. Collectively, our study suggests that excitatory and inhibitory neurons serve as the core cell populations driving the molecular mechanisms underlying cingulate functional gradient alterations, and may further contribute to the neurodegenerative pathological mechanisms that underlie brain dysfunction in patients with ESRD.

### 4.4 Limitations

Several issues have to be considered. First, we cannot rule out the potential effect of medications (e.g., antihypertensives [62], erythropoietin [63]) on the brain, which are necessary for ESRD patients. Second, the remarkable public AHBA gene expression data were derived postmortem from six cognitively normal individuals. This limitation restricts the investigation of transcriptome–neuroimaging associations across groups and likely excludes the assessment of individual‐specific effects.

## 5 Conclusion

Our findings highlight imbalances in the hierarchical organization of the cingulate functional connectome in patients with ESRD and its linkage with gene expression profiles and clinical phenotypes, offering critical insights into the neurodegenerative underpinnings of cerebral dysfunction in ESRD.

## Author Contributions

Ming Zhang and Wen Wang: conceptualisation, supervision, funding acquisition, writing – review and editing. Peng Li: conceptualisation, data curation, formal analysis, investigation, methodology, writing – original draft. Jun‐Ya Mu, Xin‐Yi Zhu, Hui‐Jie Yuan, Zhao‐Yao Luo, Qian‐Ge Zhu, Xuan Niu: data collection. Xiu‐Long Feng, Yu Han, Ting Ge, Chen‐Xi Wang: validation, visualization. All authors read and approved the final manuscript.

## Funding

This work was supported by the National Natural Science Foundation of China (82202121), Health Research and Innovation Capacity Strengthening Platform Program of Shaanxi Province (2023PT‐09), 7T MRI Precision Neurology Platform of Shaanxi Province and Innovative Team for Early Warning and Rehabilitation of Mental Fatigue (2025PT‐08), Hovering Program of Fourth Military Medical University (axjhww), Talent Foundation of Tangdu Hospital (2018BJ003), Science and Technology Research Project of Shaanxi Nuclear Industry Group Co. Ltd. (61240302), and Clinical Research Award of the First Affiliated Hospital of Xi'an Jiaotong University (XJTU1AF‐CRF‐2023‐021).

## Ethics Statement

This study was approved by the Research Ethics Committee of the First Affiliated Hospital of Xi'an Jiaotong University (Approval No. 2020G64). All procedures were conducted per the Declaration of Helsinki, and each participant provided written informed consent.

## Conflicts of Interest

The authors declare no conflicts of interest.

## Supporting information


**Figure S1:** The averaged explained ratio of the first 10 diffusion embedding components in the HCs and ESRD groups.
**Table S1:** Between‐group differences map of cingulate functional gradient.
**Table S2:** Between‐group differences in the global and subnetworks gradient metrics of the cingulate cortex.
**Table S3:** Spatial correlation between ESRD‐related alterations in the gradient 1 and the meta‐analytic map of behavioral terms.
**Table S4:** Spatial correlation between ESRD‐related alterations in the gradient 3 and the meta‐analytic map of behavioral terms.
**Table S5:** Donor information for the Allen Human Brain Atlas.
**Table S6:** Top 20 clusters with their representative enriched terms (one per cluster) based on 2231 PLS1+ genes.
**Table S7:** Top 20 clusters with their representative enriched terms (one per cluster) based on 479 PLS1− genes.

## Data Availability

The discovery data and main analysis codes that support the findings are publicly available in the study's Open Science Framework repository (https://osf.io/f2t9j/). Diffusion embedding code is publicly accessible in the BrainSpace toolbox (https://github.com/MICA‐MNI/BrainSpace). Transcriptional level matrices were obtained using the abagen toolbox (v.0.1.4, https://github.com/rmarkello/abagen) on the AHBA dataset (http://human.brain‐map.org). The probe‐to‐gene annotations were obtained by the Re‐annotator toolkit (v1.0.0, https://sourceforge.net/projects/reannotator/). Gene enrichments were analyzed at https://metascape.org/gp/index.html#/main/step1.

## References

[1] D. Viggiano , C. A. Wagner , G. Martino , et al., “Mechanisms of Cognitive Dysfunction in CKD,” Nature Reviews Nephrology 16 (2020): 452–469, 10.1038/s41581-020-0266-9. 32235904

[2] S. Zimmermann , A. Mathew , O. Bondareva , et al., “Chronic Kidney Disease Leads to Microglial Potassium Efflux and Inflammasome Activation in the Brain,” Kidney International 106 (2024): 1101–1116, 10.1016/j.kint.2024.06.028. 39089576

[3] T. D. Andrews , G. S. Day , S. R. Irani , T. Kanekiyo , and L. J. Hickson , “Uremic Toxins, CKD, and Cognitive Dysfunction,” Journal of the American Society of Nephrology 36 (2025): 1208–1226, 10.1681/ASN.0000000675. 40009460 PMC12147973

[4] A. Mathur , J. Y. B. Ahn , W. Sutton , et al., “Secondary Hyperparathyroidism (CKD‐MBD) Treatment and the Risk of Dementia,” Nephrology, Dialysis, Transplantation 37 (2022): 2111–2118, 10.1093/ndt/gfac167. PMC9585471 35512551

[5] M. Barbieri , P. Chiodini , P. di Gennaro , et al., “Efficacy of Erythropoietin as a Neuroprotective Agent in CKD‐Associated Cognitive Dysfunction: A Literature Systematic Review,” Pharmacological Research 203 (2024): 107146, 10.1016/j.phrs.2024.107146. 38493928

[6] J. S. Scherer , S. A. Combs , and F. Brennan , “Sleep Disorders, Restless Legs Syndrome, and Uremic Pruritus: Diagnosis and Treatment of Common Symptoms in Dialysis Patients,” American Journal of Kidney Diseases 69 (2017): 117–128, 10.1053/j.ajkd.2016.07.031. 27693261 PMC5497466

[7] E. Lambourg , L. Colvin , G. Guthrie , et al., “The Prevalence of Pain Among Patients With Chronic Kidney Disease Using Systematic Review and Meta‐Analysis,” Kidney International 100 (2021): 636–649, 10.1016/j.kint.2021.03.041. 33940112

[8] L. P. Gregg , M. Bossola , M. Ostrosky‐Frid , and S. S. Hedayati , “Fatigue in CKD: Epidemiology, Pathophysiology, and Treatment,” Clinical Journal of the American Society of Nephrology 16 (2021): 1445–1455, 10.2215/CJN.19891220. 33858827 PMC8729574

[9] J. E. Flythe , T. Hilliard , G. Castillo , et al., “Symptom Prioritization Among Adults Receiving In‐Center Hemodialysis: A Mixed Methods Study,” Clinical Journal of the American Society of Nephrology 13 (2018): 735–745, 10.2215/CJN.10850917. 29559445 PMC5969481

[10] C. Yu , Y. Zhou , Y. Liu , et al., “Functional Segregation of the Human Cingulate Cortex Is Confirmed by Functional Connectivity Based Neuroanatomical Parcellation,” NeuroImage 54 (2011): 2571–2581, 10.1016/j.neuroimage.2010.11.018. 21073967

[11] M. Habermann , A. Strube , and C. Büchel , “How Control Modulates Pain,” Trends in Cognitive Sciences 29 (2025): 60–72, 10.1016/j.tics.2024.09.014. 39462693

[12] P. Li , J. Mu , X. Ma , et al., “Neurovascular Coupling Dysfunction in End‐Stage Renal Disease Patients Related to Cognitive Impairment,” Journal of Cerebral Blood Flow and Metabolism 41 (2021): 2593–2606, 10.1177/0271678X211007960. 33853410 PMC8504946

[13] J. Mu , T. Chen , P. Li , et al., “Altered White Matter Microstructure Mediates the Relationship Between Hemoglobin Levels and Cognitive Control Deficits in End‐Stage Renal Disease Patients,” Human Brain Mapping 39 (2018): 4766–4775, 10.1002/hbm.24321. 30062855 PMC6866371

[14] P. Li , N. Li , L. Ren , et al., “Brain Connectome Gradient Dysfunction in Patients With End‐Stage Renal Disease and Its Association With Clinical Phenotype and Cognitive Deficits,” Communications Biology 8 (2025): 701, 10.1038/s42003-025-08132-6. 40325140 PMC12052779

[15] J. W. Vogel , R. la Joie , M. J. Grothe , et al., “A Molecular Gradient Along the Longitudinal Axis of the Human Hippocampus Informs Large‐Scale Behavioral Systems,” Nature Communications 11 (2020): 960, 10.1038/s41467-020-14518-3. PMC7031290 32075960

[16] D. S. Margulies , S. S. Ghosh , A. Goulas , et al., “Situating the Default‐Mode Network Along a Principal Gradient of Macroscale Cortical Organization,” Proceedings of the National Academy of Sciences of the United States of America 113 (2016): 12574–12579, 10.1073/pnas.1608282113. 27791099 PMC5098630

[17] R. Vos de Wael , O. Benkarim , C. Paquola , et al., “BrainSpace: A Toolbox for the Analysis of Macroscale Gradients in Neuroimaging and Connectomics Datasets,” Communications Biology 3 (2020): 103, 10.1038/s42003-020-0794-7. 32139786 PMC7058611

[18] Y. Shen , H. Cai , F. Mo , S. Yao , Y. Yu , and J. Zhu , “Functional Connectivity Gradients of the Cingulate Cortex,” Communications Biology 6 (2023): 650, 10.1038/s42003-023-05029-0. 37337086 PMC10279697

[19] C. Zoccali , F. Mallamaci , C. A. Wagner , et al., “Genetic and Circulating Biomarkers of Cognitive Dysfunction and Dementia in CKD,” Nephrology, Dialysis, Transplantation 40 (2025): ii64–ii75, 10.1093/ndt/gfae259. PMC11905751 40080085

[20] X. Kang , J. Si , J. Zhang , et al., “Combined Transcriptomic and Proteomic Profiling of the Mouse Anterior Cingulate Cortex Identifies Potential Therapeutic Targets for Pulpitis‐Induced Pain,” ACS Omega 9 (2024): 5972–5984, 10.1021/acsomega.3c09759. 38343959 PMC10851247

[21] X. Li , S. Bu , H. Pang , et al., “Mapping Striatal Functional Gradients and Associated Gene Expression in Parkinson's Disease With Continuous Cognitive Impairment,” NPJ Parkinson's Disease 11 (2025): 138, 10.1038/s41531-025-01002-2. PMC12117062 40425604

[22] Y. Li , G. Zhou , J. Peng , et al., “White Matter Dysfunction in Alzheimer's Disease Is Associated With Disease‐Related Transcriptomic Signatures,” Communications Biology 8 (2025): 820, 10.1038/s42003-025-08177-7. 40437109 PMC12120127

[23] M. J. Hawrylycz , E. S. Lein , A. L. Guillozet‐Bongaarts , et al., “An Anatomically Comprehensive Atlas of the Adult Human Brain Transcriptome,” Nature 489 (2012): 391–399, 10.1038/nature11405. 22996553 PMC4243026

[24] M. Zhu , Y. Chen , J. Zheng , et al., “Over‐Integration of Visual Network in Major Depressive Disorder and Its Association With Gene Expression Profiles,” Translational Psychiatry 15 (2025): 86, 10.1038/s41398-025-03265-y. 40097427 PMC11914485

[25] T. Yarkoni , R. A. Poldrack , T. E. Nichols , D. C. Van Essen , and T. D. Wager , “Large‐Scale Automated Synthesis of Human Functional Neuroimaging Data,” Nature Methods 8 (2011): 665–670, 10.1038/nmeth.1635. 21706013 PMC3146590

[26] B. T. T. Yeo , F. M. Krienen , J. Sepulcre , et al., “The Organization of the Human Cerebral Cortex Estimated by Intrinsic Functional Connectivity,” Journal of Neurophysiology 106 (2011): 1125–1165, 10.1152/jn.00338.2011. 21653723 PMC3174820

[27] J. T. Daugirdas , “Kt/V (and Especially Its Modifications) Remains a Useful Measure of Hemodialysis Dose,” Kidney International 88 (2015): 466–473, 10.1038/ki.2015.204. 26176827

[28] O. Esteban , C. J. Markiewicz , R. W. Blair , et al., “fMRIPrep: A Robust Preprocessing Pipeline for Functional MRI,” Nature Methods 16 (2019): 111–116, 10.1038/s41592-018-0235-4. 30532080 PMC6319393

[29] R. Ciric , A. F. G. Rosen , G. Erus , et al., “Mitigating Head Motion Artifact in Functional Connectivity MRI,” Nature Protocols 13 (2018): 2801–2826, 10.1038/s41596-018-0065-y. 30446748 PMC8161527

[30] T. D. Satterthwaite , M. A. Elliott , R. T. Gerraty , et al., “An Improved Framework for Confound Regression and Filtering for Control of Motion Artifact in the Preprocessing of Resting‐State Functional Connectivity Data,” NeuroImage 64 (2013): 240–256, 10.1016/j.neuroimage.2012.08.052. 22926292 PMC3811142

[31] L. Fan , H. Li , J. Zhuo , et al., “The Human Brainnetome Atlas: A New Brain Atlas Based on Connectional Architecture,” Cerebral Cortex 26 (2016): 3508–3526, 10.1093/cercor/bhw157. 27230218 PMC4961028

[32] R. R. Coifman , S. Lafon , A. B. Lee , et al., “Geometric Diffusions as a Tool for Harmonic Analysis and Structure Definition of Data: Diffusion Maps,” Proceedings of the National Academy of Sciences of the United States of America 102 (2005): 7426–7431, 10.1073/pnas.0500334102. 15899970 PMC1140422

[33] S. J. Hong , R. Vos de Wael , R. A. I. Bethlehem , et al., “Atypical Functional Connectome Hierarchy in Autism,” Nature Communications 10 (2019): 1022, 10.1038/s41467-019-08944-1. PMC6399265 30833582

[34] A. Arnatkeviciute , B. D. Fulcher , and A. Fornito , “A Practical Guide to Linking Brain‐Wide Gene Expression and Neuroimaging Data,” NeuroImage 189 (2019): 353–367, 10.1016/j.neuroimage.2019.01.011. 30648605

[35] R. D. Markello , A. Arnatkeviciute , J. B. Poline , B. D. Fulcher , A. Fornito , and B. Misic , “Standardizing Workflows in Imaging Transcriptomics With the Abagen Toolbox,” eLife 10 (2021): e72129, 10.7554/eLife.72129. 34783653 PMC8660024

[36] Y. Zhou , B. Zhou , L. Pache , et al., “Metascape Provides a Biologist‐Oriented Resource for the Analysis of Systems‐Level Datasets,” Nature Communications 10 (2019): 1523, 10.1038/s41467-019-09234-6. PMC6447622 30944313

[37] J. Li , J. Seidlitz , J. Suckling , et al., “Cortical Structural Differences in Major Depressive Disorder Correlate With Cell Type‐Specific Transcriptional Signatures,” Nature Communications 12 (2021): 1647, 10.1038/s41467-021-21943-5. PMC7955076 33712584

[38] J. Seidlitz , A. Nadig , S. Liu , et al., “Transcriptomic and Cellular Decoding of Regional Brain Vulnerability to Neurogenetic Disorders,” Nature Communications 11 (2020): 3358, 10.1038/s41467-020-17051-5. PMC7335069 32620757

[39] J. M. Huntenburg , P. L. Bazin , and D. S. Margulies , “Large‐Scale Gradients in Human Cortical Organization,” Trends in Cognitive Sciences 22 (2018): 21–31, 10.1016/j.tics.2017.11.002. 29203085

[40] R. L. Buckner and F. M. Krienen , “The Evolution of Distributed Association Networks in the Human Brain,” Trends in Cognitive Sciences 17 (2013): 648–665, 10.1016/j.tics.2013.09.017. 24210963

[41] S. Luo , R. F. Qi , J. Q. Wen , et al., “Abnormal Intrinsic Brain Activity Patterns in Patients With End‐Stage Renal Disease Undergoing Peritoneal Dialysis: A Resting‐State Functional MR Imaging Study,” Radiology 278 (2016): 181–189, 10.1148/radiol.2015141913. 26053309

[42] D. Ding , P. Li , X. Y. Ma , et al., “The Relationship Between Putamen‐SMA Functional Connectivity and Sensorimotor Abnormality in ESRD Patients,” Brain Imaging and Behavior 12 (2018): 1346–1354, 10.1007/s11682-017-9808-6. 29234958

[43] R. Hu , L. Gao , P. Chen , X. Wei , X. Wu , and H. Xu , “Macroscale Neurovascular Coupling and Functional Integration in End‐Stage Renal Disease Patients With Cognitive Impairment: A Multimodal MRI Study,” Journal of Neuroscience Research 102 (2024): e25277, 10.1002/jnr.25277. 38284834

[44] P. Li , S. Ma , X. Ma , et al., “Reversal of Neurovascular Decoupling and Cognitive Impairment in Patients With End‐Stage Renal Disease During a Hemodialysis Session: Evidence From a Comprehensive fMRI Analysis,” Human Brain Mapping 44 (2022): 989–1001, 10.1002/hbm.26122. 36269166 PMC9875915

[45] S. Turker , P. Kuhnke , S. B. Eickhoff , S. Caspers , and G. Hartwigsen , “Cortical, Subcortical, and Cerebellar Contributions to Language Processing: A Meta‐Analytic Review of 403 Neuroimaging Experiments,” Psychological Bulletin 149 (2023): 699–723, 10.1037/bul0000403. 37768610

[46] A. Fragueiro , A. Tosoni , F. Santacroce , et al., “The Retrosplenial Complex as an Integration Zone Between Self‐ and Map‐Based Components of Spatial Navigation and Declarative Memory: An Activation Likelihood Estimation Metanalysis,” Neuroscience and Biobehavioral Reviews 180 (2025): 106470, 10.1016/j.neubiorev.2025.106470. 41218719

[47] C. D. Giannaki , G. M. Hadjigeorgiou , C. Karatzaferi , M. C. Pantzaris , I. Stefanidis , and G. K. Sakkas , “Epidemiology, Impact, and Treatment Options of Restless Legs Syndrome in End‐Stage Renal Disease Patients: An Evidence‐Based Review,” Kidney International 85 (2014): 1275–1282, 10.1038/ki.2013.394. 24107848

[48] M. J. Koren , H. M. Blumen , E. I. Ayers , J. Verghese , and M. K. Abramowitz , “Cognitive Dysfunction and Gait Abnormalities in CKD,” Clinical Journal of the American Society of Nephrology 16 (2021): 694–704, 10.2215/CJN.16091020. 33824156 PMC8259490

[49] U. Khan , S. Rotenberg , J. I. Cameron , M. J. Oliver , and J. Farragher , “Assessing Functional Cognition in People on Maintenance Hemodialysis Using Performance‐Based Testing,” Kidney360 7 (2025): 613–623, 10.34067/KID.0000000984. 41910489 PMC13065187

[50] C. W. McIntyre and A. Jain , “Dialysis and Cognitive Impairment,” Nature Reviews Nephrology 21 (2025): 553–564, 10.1038/s41581-025-00960-3. 40275017

[51] R. Vanholder , T. Gryp , and G. Glorieux , “Urea and Chronic Kidney Disease: The Comeback of the Century? (in Uraemia Research),” Nephrology, Dialysis, Transplantation 33 (2018): 4–12, 10.1093/ndt/gfx039. 28407121

[52] N. Massey , S. S. Vasanthi , L. G. Gimenez‐Lirola , H. Tyler , and T. Thippeswamy , “Proinflammatory Cytokines, Oxidative Stress, and Organ Function as Biomarkers of Soman (GD) Chronic Neurotoxicity,” Scientific Reports 15 (2025): 9021, 10.1038/s41598-025-94190-z. 40089647 PMC11910520

[53] Q. Faucher , T. K. van der Made , E. De Lange , and R. Masereeuw , “Blood‐Brain Barrier Perturbations by Uremic Toxins: Key Contributors in Chronic Kidney Disease‐Induced Neurological Disorders?,” European Journal of Pharmaceutical Sciences 187 (2023): 106462, 10.1016/j.ejps.2023.106462. 37169097

[54] H. Wang , B. Huang , W. Wang , et al., “High Urea Induces Depression and LTP Impairment Through mTOR Signalling Suppression Caused by Carbamylation,” eBioMedicine 48 (2019): 478–490, 10.1016/j.ebiom.2019.09.049. 31628020 PMC6838447

[55] M. D'Apolito , A. L. Colia , M. Lasalvia , et al., “Urea‐Induced ROS Accelerate Senescence in Endothelial Progenitor Cells,” Atherosclerosis 263 (2017): 127–136, 10.1016/j.atherosclerosis.2017.06.028. 28641152

[56] J. L. Babitt and H. Y. Lin , “Mechanisms of Anemia in CKD,” Journal of the American Society of Nephrology 23 (2012): 1631–1634. 22935483 10.1681/ASN.2011111078 PMC3458456

[57] J.‐M. Chillon , Z. A. Massy , and B. Stengel , “Neurological Complications in Chronic Kidney Disease Patients,” Nephrology, Dialysis, Transplantation 31 (2016): 1606–1614, 10.1093/ndt/gfv315. 26359201

[58] D. M. Wilson , M. R. Cookson , L. Van Den Bosch , H. Zetterberg , D. M. Holtzman , and I. Dewachter , “Hallmarks of Neurodegenerative Diseases,” Cell 186 (2023): 693–714, 10.1016/j.cell.2022.12.032. 36803602

[59] F. Maestú , W. de Haan , M. A. Busche , and J. DeFelipe , “Neuronal Excitation/Inhibition Imbalance: Core Element of a Translational Perspective on Alzheimer Pathophysiology,” Ageing Research Reviews 69 (2021): 101372, 10.1016/j.arr.2021.101372. 34029743

[60] J. C. Lauterborn , P. Scaduto , C. D. Cox , et al., “Increased Excitatory to Inhibitory Synaptic Ratio in Parietal Cortex Samples From Individuals With Alzheimer's Disease,” Nature Communications 12 (2021): 2603, 10.1038/s41467-021-22742-8. PMC8110554 33972518

[61] G. Natale , V. Calabrese , G. Marino , et al., “Effects of Uremic Toxins on Hippocampal Synaptic Transmission: Implication for Neurodegeneration in Chronic Kidney Disease,” Cell Death Discov 7 (2021): 295, 10.1038/s41420-021-00685-9. 34657122 PMC8520534

[62] W. H. Birkenhager and J. A. Staessen , “Antihypertensives for Prevention of Alzheimer's Disease,” Lancet Neurology 5 (2006): 466–468, 10.1016/S1474-4422(06)70453-7. 16713914

[63] S. T. Lee , K. Chu , J. E. Park , et al., “Erythropoietin Improves Memory Function With Reducing Endothelial Dysfunction and Amyloid‐Beta Burden in Alzheimer's Disease Models,” Journal of Neurochemistry 120 (2012): 115–124, 10.1111/j.1471-4159.2011.07534.x. 22004348

