# Supplementary material for: Cingulate Gradient Dysfunction in End‐Stage Renal Disease: Associations With Clinical Phenotypes and Exploratory Transcriptomic Signatures
Source: CNS Neurosci Ther. 2026 Jul 1;32(7):e70976. doi: 10.1002/cns.70976 (PMC13320367; doi:10.1002/cns.70976)
Supplement: Supplementary file 1 — Figure S1: The averaged explained ratio of the first 10 diffusion embedding components in the HCs and ESRD groups. Table S1: Between‐group differences map of cingulate functional gradient. Table S2: Between‐group differences in the global and subnetworks gradient metrics of the cingulate cortex. Table S3: Spatial correlation between ESRD‐related alterations in the gradient 1 and the meta‐analytic map of behavioral terms. Table S4: Spatial correlation between ESRD‐related alterations in the gradient 3 and the meta‐analytic map of behavioral terms. Table S5: Donor information for the Allen Human Brain Atlas. Table S6: Top 20 clusters with their representative enriched terms (one per cluster) based on 2231 PLS1+ genes. Table S7: Top 20 clusters with their representative enriched terms (one per cluster) based on 479 PLS1− genes. [file CNS-32-e70976-s001.docx]

**Cingulate gradient dysfunction in end-stage renal disease: Associations with clinical phenotypes and exploratory transcriptomic signatures**

**Supplementary Materials**

**Supplementary Methods**

***Anatomical data preprocessing***

Preprocessing of anatomical and functional data was performed using fMRIPrep^1^ (v23.1.4) and the eXtensible Connectivity Pipeline (XCP)^2,3^. T1-weighted images from the input BIDS dataset were identified, with intensity non-uniformity (INU) corrected using N4BiasFieldCorrection^4^, (distributed with ANTs v2.5.0)^5^, and these served as the T1w reference throughout the workflow. The T1-weighted reference then underwent skull stripping via a Nipype implementation of the antsBrainExtraction.sh workflow from ANTs. Cerebrospinal fluid (CSF), gray matter (GM), and white matter (WM) were segmented from the skull-stripped T1w images using FAST (FSL)^6^. Brain surfaces were reconstructed using recon-all^7^(FreeSurfer v7.3.2). The previously estimated brain mask was refined using a custom adaptation of the Mindboggle^8^ method (<http://mindboggle.info>), which reconciles ANTs-derived and FreeSurfer-derived segmentations of cortical GM. Vertex-based spatial normalization to standard space (MNI152NLin6Asym) was performed via nonlinear registration using antsRegistration (ANTs v2.5.0), with brain-extracted versions of both the T1-weighted reference and T1-weighted template employed for alignment. Grayordinate “dscalar” files (containing 91k samples) were additionally resampled to the fsLR space using Connectome Workbench^9^.

***Functional data preprocessing***

For each BOLD run identified per subject, preprocessing proceeded as follows. First, a reference volume was generated using a custom fMRIPrep pipeline. Head motion parameters relative to this BOLD reference—including transformation matrices and six degrees of freedom (three rotations, three translations)—were estimated via mcflirt^10^ (FSL) prior to any spatiotemporal filtering. The BOLD reference was then co-registered to the T1w reference using bbregister^11^, with six degrees of freedom configured for the co-registration. Several confounding time series were calculated from the preprocessed BOLD data, including framewise displacement (FD), differential variation in signals (DVARS), and three region-wise global signals. FD was computed using two formulations as described by Power^12^ and Jenkinson^10^. The three global signals were extracted from the WM, CSF, and whole-brain masks. FD and DVARS were calculated for each functional run using their respective implementations in Nipype^12^. Additionally, a set of physiological regressors were extracted to enable component-based noise correction, including two CompCor^13^ variants: temporal CompCor and anatomical CompCor. The confound time series derived from head motion estimates and global signals were expanded to include temporal derivatives and quadratic terms for each regressor. Frames exceeding thresholds of 0.5 mm for FD or 1.5 for standardized DVARS were annotated as motion outliers. For FD-based censoring, a 10% data loss threshold was applied. The resulting censored dataset retained 95% of the original frames (i.e., an average of 5% were censored). Additional nuisance time series were calculated via principal component analysis of signals within a thin voxel band along the brain edge. BOLD time series were resampled onto the left/right-symmetric “fsLR” template^9^. Grayordinate files^9^ were additionally generated using the highest-resolution fsaverage template as an intermediate standardized surface space. All resamplings were performed in a single interpolation step by composing all relevant transformations (i.e., head-motion transform matrices, susceptibility distortion correction [when available], and co-registrations to anatomical and output spaces). Vertex-based resamplings were performed using ANTs, and surface resamplings using mri_vol2surf (FreeSurfer).

***Post-processing of fMRIPrep outputs***

XCP^2^, constructed using Nipype^14^ (v1.7.0), was employed to post-process fMRIPrep outputs. For each subject’s three BOLD series, post-processing proceeded as follows: Prior to nuisance regression and data filtering, FD exceeding 0.3 mm were flagged as outliers and excluded from the nuisance regression step. Thirty-six nuisance regressors were selected from the nuisance confound matrices output by fMRIPrep, including: six motion parameters and their quadratic expansions; mean WM signal, global signal, and mean CSF signal; and temporal derivatives of these tissue signals (WM, global, CSF) as well as of the motion parameters (including their quadratic terms). These regressors were removed from the BOLD data via linear regression, implemented in Scikit-Learn (v0.24.2). Residual time series from this regression were subsequently band-pass filtered to the 0.01–0.08 Hz range. The processed BOLD data were smoothed using FSL with a Gaussian kernel (6.0 mm full-width at half-maximum [FWHM]). Vertex-level pairwise functional connectivity between the cingulate cortex and the entire cerebrum was computed, operationalized as Pearson’s correlations of each parcel’s time series.

**Supplementary Results**

***Alterations in the cingulate functional gradients in ESRD patients***

The variability in cingulate rsFC patterns explained by the functional gradients is presented in descending order (Supplementary Figure 1). Gradient 1 explained 32.9% ± 0.06% of total connectivity variance (ESRD: 32.2% ± 5.8%; HCs: 34.1% ± 5.2%), showing a radiating pattern (midcingulate [A24cd, A23c] to anterior [A32sg] and posterior [A23v, A23d] cingulate; Figure 1a). Gradient 2 explained 16.3% ± 0.03% of variance (ESRD: 16.4% ± 2.6%; HCs: 16.2% ± 2.7%), following an anterior–posterior axis (anterior [A32sg, A24rv, A32p] to posterior [A23v, A23d]; Figure 1b). Gradient 3 explained 11.9% ± 0.02% of variance (ESRD: 12.0% ± 1.7%; HCs: 11.9% ± 1.6%), highlighting differentiation of subgenual (A32sg) and caudal middle (A24cd) regions from other cingulate areas (Figure 1c).

As shown in Figure 1d–f, the cingulate functional subdivision for the sensorimotor network (SMN) occupied one extreme position along gradient 1 and farthest from the default mode network (DMN) partition at the opposing extreme, with the ventral attention network (VAN), frontoparietal network (FPN), limbic network (LN), and visual network (VN) positioned in between. In contrast, the distributions of functional networks along gradients 2 and 3 were random and overlapping. No functional subdivision corresponding to the dorsal attention network was identified, which is consistent with previous study on cingulate functional gradients based on the seven-network parcellation^15^.

For gradient 2, vertex-level group comparisons revealed decreased values in ESRD patients within L_A32sg and R_A23c (TFCE, *p* < 0.05 FWE corrected; Figure 1b, Supplementary Table 1). No group differences in gradient scores were observed at the Human Brainnetome Atlas level (FDR corrected; Figure 1h, Supplementary Table 1). At the network level, ESRD patients exhibited lower gradient scores in the DMN compared to HCs (FDR corrected; Figure 1e, Supplementary Table 1).

Regarding gradient 3, vertex-level group comparisons showed decreased values in ESRD patients within L_A23v, with increased values mainly in L_A23c and L_A24cd (TFCE, *p* < 0.05 FWE corrected; Figure 1c, Supplementary Table 1). At the Human Brainnetome Atlas level, ESRD patients had lower gradient scores in R_A23v but higher scores in L_A24cd than HCs (FDR corrected; Figure 1i, Supplementary Table 1). At the network level, ESRD patients displayed reduced gradient scores in the visual network (VN) but higher scores in the DMN compared to HCs (FDR corrected; Figure 1f, Supplementary Table 1).

**Supplementary References:**

1 Esteban, O. *et al.* fMRIPrep: a robust preprocessing pipeline for functional MRI. *Nat Methods* **16**, 111-116, doi:10.1038/s41592-018-0235-4 (2019).

2 Ciric, R. *et al.* Mitigating head motion artifact in functional connectivity MRI. *Nat Protoc* **13**, 2801-2826, doi:10.1038/s41596-018-0065-y (2018).

3 Satterthwaite, T. D. *et al.* An improved framework for confound regression and filtering for control of motion artifact in the preprocessing of resting-state functional connectivity data. *Neuroimage* **64**, 240-256, doi:10.1016/j.neuroimage.2012.08.052 (2013).

4 Tustison, N. J. *et al.* N4ITK: improved N3 bias correction. *IEEE Trans Med Imaging* **29**, 1310-1320, doi:10.1109/TMI.2010.2046908 (2010).

5 Avants, B. B., Epstein, C. L., Grossman, M. & Gee, J. C. Symmetric diffeomorphic image registration with cross-correlation: evaluating automated labeling of elderly and neurodegenerative brain. *Med Image Anal* **12**, 26-41, doi:10.1016/j.media.2007.06.004 (2008).

6 Zhang, Y., Brady, M. & Smith, S. Segmentation of brain MR images through a hidden Markov random field model and the expectation-maximization algorithm. *IEEE Trans Med Imaging* **20**, 45-57, doi:10.1109/42.906424 (2001).

7 Dale, A. M., Fischl, B. & Sereno, M. I. Cortical surface-based analysis. I. Segmentation and surface reconstruction. *Neuroimage* **9**, 179-194, doi:10.1006/nimg.1998.0395 (1999).

8 Klein, A. *et al.* Mindboggling morphometry of human brains. *PLoS Comput Biol* **13**, e1005350, doi:10.1371/journal.pcbi.1005350 (2017).

9 Glasser, M. F. *et al.* The minimal preprocessing pipelines for the Human Connectome Project. *Neuroimage* **80**, 105-124, doi:10.1016/j.neuroimage.2013.04.127 (2013).

10 Jenkinson, M., Bannister, P., Brady, M. & Smith, S. Improved optimization for the robust and accurate linear registration and motion correction of brain images. *Neuroimage* **17**, 825-841, doi:10.1016/s1053-8119(02)91132-8 (2002).

11 Greve, D. N. & Fischl, B. Accurate and robust brain image alignment using boundary-based registration. *Neuroimage* **48**, 63-72, doi:10.1016/j.neuroimage.2009.06.060 (2009).

12 Power, J. D. *et al.* Methods to detect, characterize, and remove motion artifact in resting state fMRI. *Neuroimage* **84**, 320-341, doi:10.1016/j.neuroimage.2013.08.048 (2014).

13 Behzadi, Y., Restom, K., Liau, J. & Liu, T. T. A component based noise correction method (CompCor) for BOLD and perfusion based fMRI. *Neuroimage* **37**, 90-101, doi:10.1016/j.neuroimage.2007.04.042 (2007).

14 Gorgolewski, K. *et al.* Nipype: a flexible, lightweight and extensible neuroimaging data processing framework in python. *Front Neuroinform* **5**, 13, doi:10.3389/fninf.2011.00013 (2011).

15 Shen, Y. *et al.* Functional connectivity gradients of the cingulate cortex. *Commun Biol* **6**, 650, doi:10.1038/s42003-023-05029-0 (2023).


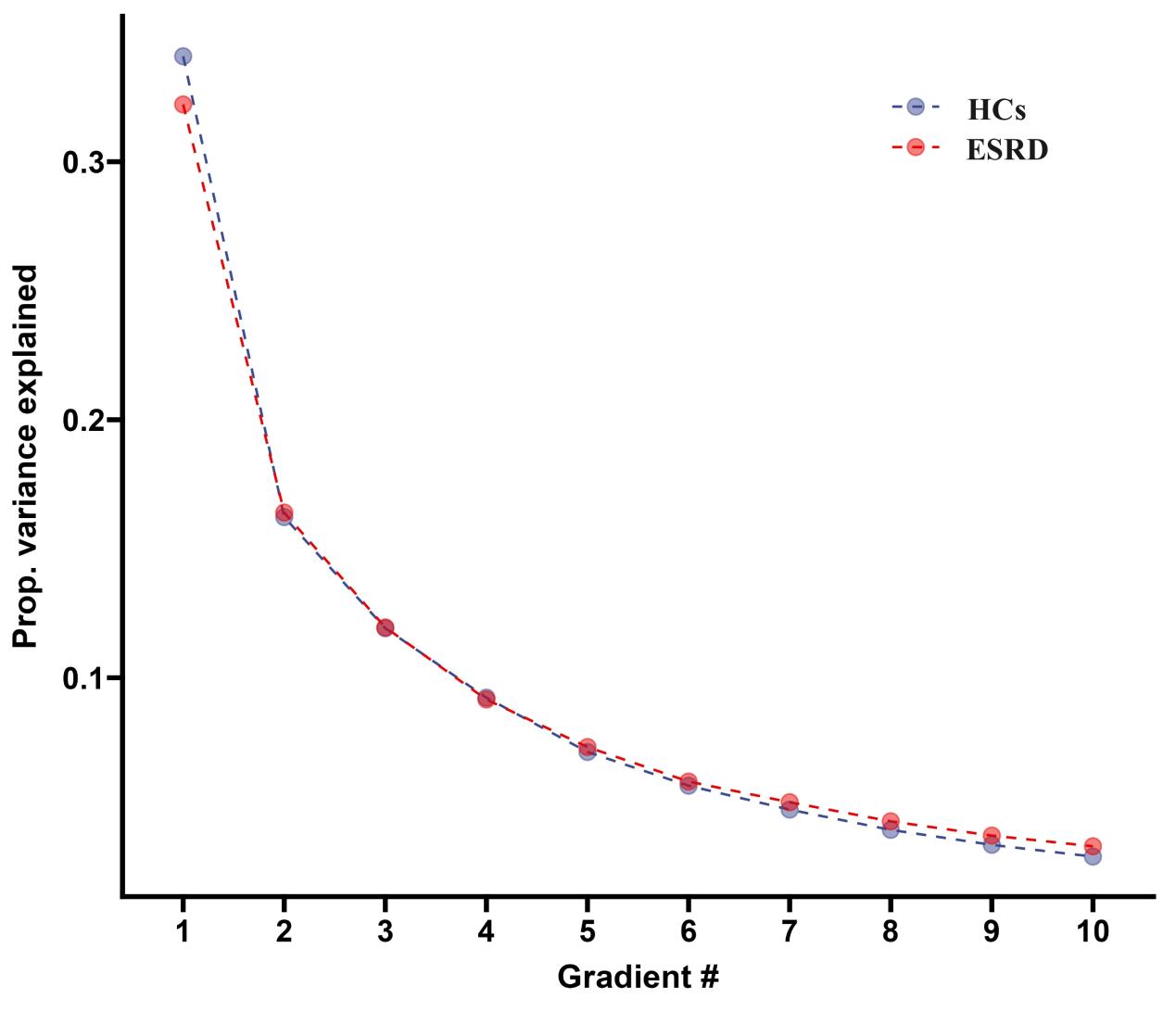


**Supplementary Fig.1** The averaged explained ratio of the first 10 diffusion embedding components in the HCs and ESRD groups. The HCs are shown in blue, and the ESRD patients are shown in red.

| **Supplementary Table 1. Between-group differences map of cingulate functional gradient (TFCE and** p<0.05 FWE). | | | | | | |
| --- | --- | --- | --- | --- | --- | --- |
| Brain Region | X | Y | Z | Left or right hemispheres | Cluster size | Sign(t) * (1-p) |
|  |  |  |  |  |  |  |
|  |  |  |  |  |  |  |
| **Gradient1: ESRD < HCs** | | | | | | |
| A23d | -3 | -34 | 39 | L | 24 | -0.9519 |
| A32sg | -10 | 46 | 6 | L | 74 | -0.9782 |
| **Gradient1: ESRD > HCs** | | | | | | |
| A24cd | -9 | 7 | 38 | L | 103 | 0.9875 |
| A23c | 11 | -26 | 40 | R | 199 | 0.9960 |
| A24cd | 10 | 5 | 40 | R | 306 | 0.9996 |
| **Gradient2: ESRD < HCs** | | | | | | |
| A32sg | -4 | 39 | 6 | L | 113 | -0.9742 |
| A23c | 4 | -21 | 41 | R | 161 | -0.9784 |
| **Gradient2: ESRD > HCs, no between-group difference** | | | | | | |
| **Gradient3: ESRD < HCs** | | | | | | |
| A23v | -22 | -54 | 4 | L | 113 | -0.9764 |
| A23v | 16 | -50 | 5 | R | 42 | -0.9684 |
| A23v | 10 | -41 | 2 | R | 20 | -0.9530 |
| **Gradient3: ESRD > HCs** | | | | | | |
| A23c | -3 | -30 | 40 | L | 216 | 0.9858 |
| A24cd | -5 | 12 | 35 | L | 56 | 0.9569 |
| Abbreviations: ESRD, end-stage renal disease; HCs, Healthy controls; TFCE, Threshold-free cluster enhancement; FWE, family-wise error. | | | | | | |
|  |  |  |  |  |  |  |

| **Supplementary Table 2. Between-group differences in the global and subnetworks gradient metrics of the cingulate cortex** | | | | | |
| --- | --- | --- | --- | --- | --- |
| **Metric** | **HCs** | **ESRD** | ***t*** | ***p*** | ***p*_FDR_** |
| **Global** | | | | | |
| G1 explained ratio | 0.340±0.052 | 0.322±0.058 | -0.927 | 0.356 | 0.649 |
| G2 explained ratio | 0.162±0.027 | 0.164±0.026 | -0.067 | 0.947 | 0.947 |
| G3 explained ratio | 0.119±0.016 | 0.120±0.017 | -0.787 | 0.433 | 0.649 |
| G1 range | 0.357±0.045 | 0.315±0.057 | -3.405 | 0.001 | **0.003** |
| G2 range | 0.192±0.036 | 0.179±0.035 | -2.338 | 0.021 | **0.021** |
| G3 range | 0.202±0.046 | 0.182±0.044 | -2.783 | 0.006 | **0.009** |
| G1 variance | 0.116±0.021 | 0.100±0.025 | -2.872 | 0.005 | **0.014** |
| G2 variance | 0.050±0.013 | 0.046±0.012 | -1.731 | 0.086 | 0.086 |
| G3 variance | 0.045±0.013 | 0.041±0.012 | -2.133 | 0.035 | 0.052 |
| **Yeo's seven functional networks** | | | | | |
| G1 | | | | | |
| Visual network | 0.032±0.051 | 0.038±0.047 | 0.943 | 0.347 | 0.417 |
| Sensorimotor network | -0.112±0.039 | -0.090±0.040 | 1.842 | 0.068 | 0.136 |
| Ventral attention network | -0.103±0.023 | -0.083±0.028 | 3.266 | 0.001 | **0.004** |
| Limbic network | 0.011±0.044 | 0.003±0.040 | -1.244 | 0.216 | 0.324 |
| Frontoparietal network | 0.003±0.036 | 0.005±0.032 | 0.252 | 0.802 | 0.802 |
| Default mode network | 0.090±0.019 | 0.071±0.022 | -3.985 | <0.001 | **0.001** |
| G2 |  |  |  |  |  |
| Visual network | -0.056±0.029 | -0.045±0.028 | 2.231 | 0.028 | 0.083 |
| Sensorimotor network | -0.023±0.026 | -0.013±0.021 | 1.962 | 0.052 | 0.102 |
| Ventral attention network | 0.008±0.008 | -0.010±0.006 | 1.842 | 0.068 | 0.102 |
| Limbic network | 0.040±0.027 | 0.044±0.032 | 0.257 | 0.798 | 0.798 |
| Frontoparietal network | 0.004±0.015 | 0.006±0.015 | 1.277 | 0.204 | 0.245 |
| Default mode network | -0.001±0.006 | -0.004±0.005 | -3.749 | <0.001 | **0.002** |
| G3 |  |  |  |  |  |
| Visual network | 0.113±0.040 | 0.087±0.038 | -3.898 | <0.001 | **0.001** |
| Sensorimotor network | 0.001±0.017 | 0.001±0.016 | -0.928 | 0.355 | 0.426 |
| Ventral attention network | -0.007±0.004 | -0.006±0.005 | 2.268 | 0.025 | 0.051 |
| Limbic network | 0.037±0.025 | 0.032±0.024 | -1.064 | 0.289 | 0.426 |
| Frontoparietal network | -0.014±0.012 | -0.014±0.015 | 0.325 | 0.746 | 0.746 |
| Default mode network | -0.011±0.007 | -0.009±0.006 | 2.495 | 0.014 | **0.042** |
| **Human Brainnetome Atlas subregions** | | | | | |
| G1 | | | | | |
| L_A23d | 0.134±0.030 | 0.119±0.035 | -1.906 | 0.059 | 0.103 |
| L_A24rv | -0.009±0.045 | -0.030±0.029 | -2.865 | 0.005 | **0.045** |
| L_A32p | -0.034±0.056 | -0.050±0.041 | -1.961 | 0.052 | 0.103 |
| L_A23v | 0.084±0.038 | 0.079±0.040 | -0.215 | 0.830 | 0.830 |
| L_A24cd | -0.115±0.036 | -0.094±0.032 | 2.775 | 0.006 | **0.045** |
| L_A23c | -0.060±0.042 | -0.056±0.035 | 0.378 | 0.706 | 0.760 |
| L_A32sg | 0.038±0.043 | 0.019±0.042 | -2.278 | 0.024 | 0.069 |
| R_A23d | 0.129±0.033 | 0.117±0.033 | -1.362 | 0.176 | 0.246 |
| R_A24rv | -0.027±0.037 | -0.028±0.030 | -0.582 | 0.562 | 0.655 |
| R_A32p | -0.105±0.042 | 0.089±0.038 | 1.633 | 0.105 | 0.163 |
| R_A23v | 0.076±0.033 | 0.079±0.037 | 1.206 | 0.230 | 0.293 |
| R_A24cd | -0.107±0.040 | -0.085±0.036 | 2.510 | 0.013 | **0.047** |
| R_A23c | -0.049±0.032 | -0.033±0.043 | 2.007 | 0.047 | 0.103 |
| R_A32sg | 0.027±0.048 | 0.003±0.042 | -2.571 | 0.011 | **0.047** |
| G2 | | | | | |
| L_A23d | -0.028±0.018 | -0.029±0.016 | -0.419 | 0.676 | 0.728 |
| L_A24rv | 0.031±0.023 | 0.031±0.022 | 0.800 | 0.425 | 0.541 |
| L_A32p | 0.064±0.023 | 0.052±0.021 | -2.629 | 0.010 | 0.136 |
| L_A23v | -0.053±0.021 | -0.047±0.023 | 2.043 | 0.043 | 0.202 |
| L_A24cd | 0.009±0.017 | 0.013±0.019 | 1.611 | 0.110 | 0.282 |
| L_A23c | -0.012±0.014 | -0.009±0.019 | 0.945 | 0.346 | 0.485 |
| L_A32sg | 0.053±0.027 | 0.050±0.031 | -1.378 | 0.171 | 0.334 |
| R_A23d | -0.029±0.019 | -0.030±0.015 | -0.655 | 0.514 | 0.599 |
| R_A24rv | 0.029±0.016 | 0.030±0.018 | 1.191 | 0.236 | 0.367 |
| R_A32p | 0.041±0.023 | 0.036±0.021 | -1.315 | 0.191 | 0.334 |
| R_A23v | -0.048±0.016 | -0.043±0.020 | 1.562 | 0.121 | 0.282 |
| R_A24cd | -0.011±0.020 | 0.017±0.023 | 1.673 | 0.097 | 0.282 |
| R_A23c | -0.006±0.014 | -0.005±0.018 | 0.189 | 0.850 | 0.850 |
| R_A32sg | 0.061±0.024 | 0.054±0.026 | -2.256 | 0.026 | 0.181 |
| G3 | | | | | |
| L_A23d | -0.036±0.016 | -0.031±0.016 | 2.220 | 0.028 | 0.071 |
| L_A24rv | 0.008±0.013 | 0.001±0.017 | 2.534 | 0.013 | 0.059 |
| L_A32p | 0.011±0.014 | 0.009±0.013 | 0.106 | 0.916 | 0.943 |
| L_A23v | 0.062±0.028 | 0.051±0.028 | -2.423 | 0.017 | 0.059 |
| L_A24cd | -0.008±0.011 | -0.002±0.012 | 3.039 | 0.003 | **0.021** |
| L_A23c | -0.015±0.011 | -0.011±0.012 | 1.221 | 0.224 | 0.297 |
| L_A32sg | 0.030±0.018 | 0.026±0.020 | -1.198 | 0.233 | 0.297 |
| R_A23d | -0.034±0.017 | -0.031±0.016 | 1.284 | 0.202 | 0.297 |
| R_A24rv | 0.009±0.012 | 0.008±0.018 | -0.352 | 0.725 | 0.846 |
| R_A32p | 0.001±0.010 | 0.004±0.014 | 1.632 | 0.105 | 0.211 |
| R_A23v | 0.047±0.020 | 0.036±0.023 | -3.026 | 0.003 | **0.021** |
| R_A24cd | -0.009±0.012 | -0.004±0.017 | 1.299 | 0.196 | 0.297 |
| R_A23c | -0.019±0.009 | -0.020±0.014 | -0.072 | 0.943 | 0.943 |
| R_A32sg | 0.020±0.017 | 0.015±0.016 | -2.189 | 0.031 | 0.071 |
| Abbreviations: ESRD = end-stage renal disease; HCs = Healthy controls; G1 = Gradient 1; G2 = Gradient 2; G3 = Gradient 3; FDR = false discovery rate; data have a 95% confidence interval after controlling age, sex and education level. | | | | | |

| **Supplementary Table 3. Spatial correlation between ESRD-related alterations in the gradient 1 and the meta-analytic map of behavioral terms** | | | | | | | |
| --- | --- | --- | --- | --- | --- | --- | --- |
|  |  |  |  |  |  |  |  |
| ESRD-positive | | | | ESRD-negative | | | |
| Term | *r* | *P* | P_FDR_ | Term | *r* | *P* | P_FDR_ |
| pain | 0.1657 | 0.00779844 | **0.00974805** | value | -0.0898 | 0.16756649 | 0.24349676 |
| electrical | 0.1160 | 0.00779844 | **0.00974805** | dysfunction | -0.0878 | 0.08038392 | 0.19980619 |
| autonomic | 0.1001 | 0.00779844 | **0.00974805** | dysregulation | -0.0829 | 0.08658268 | 0.19980619 |
| secondary somatosensory | 0.0938 | 0.00019996 | **0.00054535** | negative | -0.0765 | 0.14357129 | 0.24349676 |
| somatosensory | 0.0917 | 0.00019996 | **0.00054535** | regulatory | -0.0762 | 0.03359328 | 0.19414299 |
| noxious | 0.0892 | 0.00779844 | **0.00974805** | characterized | -0.0733 | 0.16536693 | 0.24349676 |
| intensity | 0.0869 | 0.00019996 | **0.00054535** | affect | -0.0732 | 0.03079384 | 0.19414299 |
| sensations | 0.0801 | 0.00779844 | **0.00974805** | reward | -0.0703 | 0.19896021 | 0.25794841 |
| circuits | 0.0797 | 0.00779844 | **0.00974805** | emotion | -0.0702 | 0.07118576 | 0.19414299 |
| aversive | 0.0761 | 0.02039592 | **0.02353375** | regulation | -0.0687 | 0.22015597 | 0.26418716 |
| target detection | 0.0748 | 0.00019996 | **0.00054535** | rewards | -0.0669 | 0.17856429 | 0.24349676 |
| conflict | 0.0743 | 0.10777844 | 0.11149494 | clarify | -0.0665 | 0.02599480 | 0.19414299 |
| sensation | 0.0738 | 0.00019996 | **0.00054535** | negative affect | -0.0652 | 0.05758848 | 0.19414299 |
| induced | 0.0728 | 0.00779844 | **0.00974805** | anxiety | -0.0638 | 0.17096581 | 0.24349676 |
| ratings | 0.0716 | 0.09038192 | 0.09683778 | affective | -0.0634 | 0.16096781 | 0.24349676 |
| nociceptive | 0.0711 | 0.01019796 | **0.01223755** | personality | -0.0625 | 0.13717257 | 0.24349676 |
| motor | 0.0690 | 0.00779844 | **0.00974805** | self | -0.0622 | 0.46190762 | 0.47783547 |
| preparation | 0.0685 | 0.00559888 | **0.00974805** | neutral | -0.0621 | 0.06138772 | 0.19414299 |
| evoked | 0.0683 | 0.00779844 | **0.00974805** | punishment | -0.0620 | 0.05998800 | 0.19414299 |
| control | 0.0682 | 0.06318736 | 0.07020818 | emotions | -0.0617 | 0.04419116 | 0.19414299 |
| sampling | 0.0681 | 0.00019996 | **0.00054535** | referential | -0.0608 | 0.34973005 | 0.38858895 |
| stimulation | 0.0679 | 0.00779844 | **0.00974805** | choices | -0.0607 | 0.24675065 | 0.28471229 |
| avoid | 0.0677 | 0.00019996 | **0.00054535** | stress disorder | -0.0607 | 0.03839232 | 0.19414299 |
| actual | 0.0671 | 0.00019996 | **0.00054535** | middle cingulate | -0.0606 | 0.66526695 | 0.66526695 |
| responses | 0.0663 | 0.11297740 | 0.11297740 | background | -0.0605 | 0.14357129 | 0.24349676 |
| event | 0.0661 | 0.00779844 | **0.00974805** | self referential | -0.0598 | 0.37372525 | 0.40041992 |
| primary somatosensory | 0.0643 | 0.00019996 | **0.00054535** | anxiety disorders | -0.0581 | 0.06678664 | 0.19414299 |
| target | 0.0639 | 0.00779844 | **0.00974805** | reinforcement | -0.0580 | 0.13157369 | 0.24349676 |
| reaching | 0.0624 | 0.00019996 | **0.00054535** | impulsivity | -0.0578 | 0.20635873 | 0.25794841 |
| sensorimotor | 0.0622 | 0.00019996 | **0.00054535** | ptsd | -0.0577 | 0.05658868 | 0.19414299 |
| Abbreviations: ESRD, end-stage renal disease; FDR, false discovery rate. | | | | | | | |

| **Supplementary Table 4. Spatial correlation between ESRD-related alterations in the gradient 3 and the meta-analytic map of behavioral terms** | | | | | | | |
| --- | --- | --- | --- | --- | --- | --- | --- |
|  |  |  |  |  |  |  |  |
| ESRD-positive | | | | ESRD-negative | | | |
| Term | *r* | *P* | *P*_FDR_ | Term | *r* | *P* | *P*_FDR_ |
| pain | 0.1266 | 0.00019996 | **0.00026082** | deactivation | -0.0791 | 0.14217157 | 0.17921416 |
| electrical | 0.0884 | 0.00019996 | **0.00026082** | autobiographical | -0.0763 | 0.07238552 | 0.10340789 |
| somatosensory | 0.0766 | 0.00019996 | **0.00026082** | episodic memory | -0.0741 | 0.07238552 | 0.10340789 |
| secondary somatosensory | 0.0761 | 0.00019996 | **0.00026082** | default | -0.0716 | 0.35812837 | 0.37047763 |
| supplementary | 0.0743 | 0.00019996 | **0.00026082** | autobiographical memory | -0.0712 | 0.07238552 | 0.10340789 |
| intensity | 0.0693 | 0.00019996 | **0.00026082** | episodic | -0.0695 | 0.14337133 | 0.17921416 |
| motor sma | 0.0689 | 0.00019996 | **0.00026082** | rest | -0.0640 | 0.00019996 | **0.00054535** |
| target detection | 0.0682 | 0.00019996 | **0.00026082** | recall | -0.0629 | 0.00019996 | **0.00054535** |
| sampling | 0.0672 | 0.00019996 | **0.00026082** | memories | -0.0610 | 0.07118576 | 0.10340789 |
| autonomic | 0.0645 | 0.07158568 | 0.07755592 | navigation | -0.0591 | 0.00019996 | **0.00054535** |
| avoid | 0.0631 | 0.00019996 | **0.00026082** | dysfunction | -0.0583 | 0.21335733 | 0.24239597 |
| noxious | 0.0627 | 0.07158568 | 0.07755592 | construction | -0.0566 | 0.00019996 | **0.00054535** |
| actual | 0.0609 | 0.00019996 | **0.00026082** | semantic memory | -0.0554 | 0.07238552 | 0.10340789 |
| conflict | 0.0589 | 0.14477105 | 0.14497101 | children adolescents | -0.0551 | 0.00019996 | **0.00054535** |
| sensation | 0.0571 | 0.00019996 | **0.00026082** | remembering | -0.0548 | 0.07238552 | 0.10340789 |
| voluntary | 0.0565 | 0.00019996 | **0.00026082** | neuropsychiatric | -0.0539 | 0.00019996 | **0.00054535** |
| primary secondary | 0.0561 | 0.00019996 | **0.00026082** | past | -0.0514 | 0.07238552 | 0.10340789 |
| distraction | 0.0558 | 0.00019996 | **0.00026082** | scenes | -0.0491 | 0.00019996 | **0.00054535** |
| nociceptive | 0.0555 | 0.00019996 | **0.00026082** | stable | -0.0490 | 0.07118576 | 0.10340789 |
| preparation | 0.0554 | 0.00019996 | **0.00026082** | disorders | -0.0483 | 0.21815637 | 0.24239597 |
| ratings | 0.0553 | 0.07238552 | 0.07755592 | memory retrieval | -0.0469 | 0.21715657 | 0.24239597 |
| responses | 0.0547 | 0.07038592 | 0.07755592 | elderly | -0.0466 | 0.00019996 | **0.00054535** |
| basic | 0.0539 | 0.00019996 | **0.00026082** | opposed | -0.0463 | 0.00019996 | **0.00054535** |
| speech production | 0.0537 | 0.00019996 | **0.00026082** | state | -0.0457 | 0.35812837 | 0.37047763 |
| sensations | 0.0528 | 0.07158568 | 0.07755592 | memory | -0.0456 | 0.14337133 | 0.17921416 |
| aversive | 0.0517 | 0.14497101 | 0.14497101 | self | -0.0456 | 0.49890022 | 0.49890022 |
| reactions | 0.0514 | 0.00019996 | **0.00026082** | recognition task | -0.0454 | 0.07118576 | 0.10340789 |
| primary sensorimotor | 0.0513 | 0.00019996 | **0.00026082** | structures | -0.0452 | 0.07238552 | 0.10340789 |
| primary somatosensory | 0.0513 | 0.00019996 | **0.00026082** | weaker | -0.0450 | 0.00019996 | **0.00054535** |
| sensorimotor | 0.0511 | 0.00019996 | **0.00026082** | impairment | -0.0436 | 0.00019996 | **0.00054535** |
| Abbreviations: ESRD, end-stage renal disease; FDR, false discovery rate. | | | | | | | |

| **Supplementary Table 5 Donor information for the Allen Human Brain Atlas.** | | | | | |
| --- | --- | --- | --- | --- | --- |
|  |  |  |  |  |  |
| Donor ID | Age | Sex | Postmortem interval | Handedness | Hemispheres |
| H0351.2001 | 24 | Male | 23 h | Left | Left + Right |
| H0351.2002 | 39 | Male | 10 h | Left | Left + Right |
| H0351.1009 | 57 | Male | 25.5 h | Cross-dominant | Left |
| H0351.1012 | 31 | Male | 17.5 h | Right | Left |
| H0351.1015 | 49 | Female | 30 h | Right | Left |
| H0351.1016 | 55 | Male | 18 h | Right | Left |

| **Supplementary table 6. Top 20 clusters with their representative enriched terms (one per cluster) based on 2231 PLS1+ genes** | | | | | | |
| --- | --- | --- | --- | --- | --- | --- |
| **GO** | **Category** | **Description** | **Count** | **%** | **Log10(*p*)** | **Log10(*q*)** |
| R-HSA-156827 | Reactome Gene Sets | L13a-mediated translational silencing of Ceruloplasmin expression | 50 | 2.27 | -26.79 | -22.52 |
| GO:0022613 | GO Biological Processes | ribonucleoprotein complex biogenesis | 88 | 4 | -14.84 | -12.07 |
| GO:0019941 | GO Biological Processes | modification-dependent protein catabolic process | 103 | 4.69 | -14.69 | -11.93 |
| GO:0007005 | GO Biological Processes | mitochondrion organization | 74 | 3.37 | -14.46 | -11.73 |
| GO:0070201 | GO Biological Processes | regulation of establishment of protein localization | 93 | 4.23 | -13.61 | -10.92 |
| GO:0051668 | GO Biological Processes | localization within membrane | 93 | 4.23 | -12.71 | -10.05 |
| R-HSA-1632852 | Reactome Gene Sets | Macroautophagy | 38 | 1.73 | -11.65 | -9 |
| hsa05022 | KEGG Pathway | Pathways of neurodegeneration - multiple diseases | 80 | 3.64 | -11.45 | -8.82 |
| GO:0042176 | GO Biological Processes | regulation of protein catabolic process | 68 | 3.09 | -11.21 | -8.6 |
| GO:0006753 | GO Biological Processes | nucleoside phosphate metabolic process | 88 | 4 | -9.82 | -7.28 |
| R-HSA-9609507 | Reactome Gene Sets | Protein localization | 37 | 1.68 | -9.48 | -6.95 |
| GO:0030097 | GO Biological Processes | hemopoiesis | 100 | 4.55 | -9.48 | -6.95 |
| R-HSA-199991 | Reactome Gene Sets | Membrane Trafficking | 91 | 4.14 | -9.41 | -6.9 |
| GO:0006886 | GO Biological Processes | intracellular protein transport | 85 | 3.87 | -9.35 | -6.85 |
| GO:0006914 | GO Biological Processes | autophagy | 61 | 2.78 | -8.81 | -6.35 |
| GO:1903320 | GO Biological Processes | regulation of protein modification by small protein conjugation or removal | 46 | 2.09 | -8.78 | -6.33 |
| GO:0050808 | GO Biological Processes | synapse organization | 61 | 2.78 | -8.76 | -6.32 |
| GO:0010564 | GO Biological Processes | regulation of cell cycle process | 103 | 4.69 | -8.71 | -6.27 |
| GO:1903828 | GO Biological Processes | negative regulation of protein localization | 45 | 2.05 | -8.59 | -6.16 |
| GO:0022618 | GO Biological Processes | protein-RNA complex assembly | 44 | 2 | -8.58 | -6.15 |
| Note: "Count" is the number of genes in the PLS1+ gene list with membership in the given ontology term. "%" is the percentage of all PLS1+ genes found in the given ontology term (only input genes with at least one ontology term annotation are included in the calculation). "Log10(*p*)" is the p-value in log base 10. "Log10(*q*)" is the multitest adjusted *p*-value in log base 10. | | | | | | |

| **Supplementary table 7. Top 20 clusters with their representative enriched terms (one per cluster) based on 479 PLS1- genes** | | | | | | |
| --- | --- | --- | --- | --- | --- | --- |
| **GO** | **Category** | **Description** | **Count** | **%** | **Log10(*p*)** | **Log10(*q*)** |
| GO:0006259 | GO Biological Processes | DNA metabolic process | 83 | 5.56 | -10.88 | -6.54 |
| GO:0032446 | GO Biological Processes | protein modification by small protein conjugation | 73 | 4.89 | -8.54 | -4.52 |
| GO:0006886 | GO Biological Processes | intracellular protein transport | 63 | 4.22 | -8.39 | -4.52 |
| WP3888 | WikiPathways | VEGFA VEGFR2 signaling | 51 | 3.42 | -8.07 | -4.34 |
| WP5475 | WikiPathways | Hallmark of cancer sustaining proliferative signaling | 21 | 1.41 | -7.98 | -4.34 |
| GO:0006351 | GO Biological Processes | DNA-templated transcription | 61 | 4.09 | -7.09 | -3.67 |
| GO:0016071 | GO Biological Processes | mRNA metabolic process | 65 | 4.36 | -6.85 | -3.51 |
| GO:0016310 | GO Biological Processes | phosphorylation | 44 | 2.95 | -6.65 | -3.35 |
| GO:0031346 | GO Biological Processes | positive regulation of cell projection organization | 42 | 2.82 | -6.39 | -3.19 |
| GO:0006338 | GO Biological Processes | chromatin remodeling | 66 | 4.42 | -6.38 | -3.19 |
| GO:0034655 | GO Biological Processes | nucleobase-containing compound catabolic process | 42 | 2.82 | -6.36 | -3.19 |
| R-HSA-199991 | Reactome Gene Sets | Membrane Trafficking | 61 | 4.09 | -6.22 | -3.1 |
| GO:0051301 | GO Biological Processes | cell division | 53 | 3.55 | -6.03 | -3.01 |
| GO:0010256 | GO Biological Processes | endomembrane system organization | 58 | 3.89 | -5.98 | -3 |
| GO:0090407 | GO Biological Processes | organophosphate biosynthetic process | 55 | 3.69 | -5.7 | -2.85 |
| GO:0051668 | GO Biological Processes | localization within membrane | 55 | 3.69 | -5.68 | -2.84 |
| GO:0048871 | GO Biological Processes | multicellular organismal-level homeostasis | 62 | 4.16 | -5.62 | -2.82 |
| GO:0099003 | GO Biological Processes | vesicle-mediated transport in synapse | 24 | 1.61 | -5.46 | -2.73 |
| WP437 | WikiPathways | EGF EGFR signaling | 23 | 1.54 | -5.44 | -2.73 |
| R-HSA-3108214 | Reactome Gene Sets | SUMOylation of DNA damage response and repair proteins | 15 | 1.01 | -5.34 | -2.65 |
| Note: "Count" is the number of genes in the PLS1- gene list with membership in the given ontology term. "%" is the percentage of all PLS1- genes found in the given ontology term (only input genes with at least one ontology term annotation are included in the calculation). "Log10(*p*)" is the p-value in log base 10. "Log10(*q*)" is the multitest adjusted *p*-value in log base 10. | | | | | | |
